# Ultrastructure of the liver microcirculation influences hepatic and systemic insulin activity and provides a mechanism for age‐related insulin resistance

**DOI:** 10.1111/acel.12481

**Published:** 2016-04-20

**Authors:** Mashani Mohamad, Sarah Jayne Mitchell, Lindsay Edward Wu, Melanie Yvonne White, Stuart James Cordwell, John Mach, Samantha Marie Solon‐Biet, Dawn Boyer, Dawn Nines, Abhirup Das, Shi‐Yun Catherine Li, Alessandra Warren, Sarah Nicole Hilmer, Robin Fraser, David Andrew Sinclair, Stephen James Simpson, Rafael de Cabo, David George Le Couteur, Victoria Carroll Cogger

**Affiliations:** ^1^ Ageing and Alzheimers InstituteCentre for Education and Research on AgeingUniversity of Sydney and Concord HospitalSydneyNSWAustralia; ^2^ ANZAC Research InstituteUniversity of Sydney and Concord HospitalSydneyNSWAustralia; ^3^ Faculty of PharmacyUniversiti Teknologi MARASelangorMalaysia; ^4^ Translational Gerontology BranchNational Institute on AgingNational Institutes of HealthBaltimoreMDUSA; ^5^ Laboratory for Ageing ResearchSchool of Medical SciencesUniversity of New South WalesSydneyNSWAustralia; ^6^ Charles Perkins CentreUniversity of SydneySydneyNSWAustralia; ^7^ Kolling Institute of Medical ResearchRoyal North Shore Hospital and University of SydneySydneyNSWAustralia; ^8^ Department of PathologyUniversity of OtagoChristchurchNew Zealand; ^9^ Department of GeneticsHarvard Medical SchoolBostonMAUSA

**Keywords:** ageing, aging, fenestrations, fenestrae, endothelium, hyperinsulinemia

## Abstract

While age‐related insulin resistance and hyperinsulinemia are usually considered to be secondary to changes in muscle, the liver also plays a key role in whole‐body insulin handling and its role in age‐related changes in insulin homeostasis is largely unknown. Here, we show that patent pores called ‘fenestrations’ are essential for insulin transfer across the liver sinusoidal endothelium and that age‐related loss of fenestrations causes an impaired insulin clearance and hyperinsulinemia, induces hepatic insulin resistance, impairs hepatic insulin signaling, and deranges glucose homeostasis. To further define the role of fenestrations in hepatic insulin signaling without any of the long‐term adaptive responses that occur with aging, we induced acute defenestration using poloxamer 407 (P407), and this replicated many of the age‐related changes in hepatic glucose and insulin handling. Loss of fenestrations in the liver sinusoidal endothelium is a hallmark of aging that has previously been shown to cause deficits in hepatic drug and lipoprotein metabolism and now insulin. Liver defenestration thus provides a new mechanism that potentially contributes to age‐related insulin resistance.

## Introduction

Insulin resistance, characterized by elevated basal and stimulated levels of insulin, is inextricably associated with older age (Fink *et al*., 1983; Oya *et al*., 2014). The liver is the key target organ for insulin activity, and under normal physiological conditions, the entire output of endogenous insulin from the pancreas travels through the liver vasculature before entering the systemic circulation (Izzo & Bartlett, 1969). Once it has entered the liver parenchyma, insulin regulates myriad metabolic processes such as glycogen storage, gluconeogenesis, and fatty acid synthesis. The primary role of the liver in the development of insulin resistance is demonstrated by the fact that insulin resistance occurs in liver‐specific insulin receptor‐knockout mice (Fisher & Kahn, 2003), but not in muscle‐ or fat‐specific insulin receptor‐knockout mice (Bruning *et al*., 1998; Bluher *et al*., 2002). Further evidence comes from the observation that glucose intolerance that occurs during the early stages of high fat feeding is due to the development of insulin resistance in the liver before other tissues develop metabolic changes (Turner *et al*., 2013). Hyperinsulinemia and insulin resistance are common in conditions associated with altered liver function, particularly those associated with structural changes in the liver endothelium such as old age and liver disease (Fink *et al*., 1983; Muller *et al*., 1992; Kotronen *et al*., 2007; Kawaguchi *et al*., 2011; Takamura *et al*., 2012; Bose & Ray, 2014; Chai *et al*., 2014; Taguchi *et al*., 2014).

The transfer of insulin across the endothelium is the rate‐limiting step for the uptake and action of insulin in the muscle and the fat (Sandqvist *et al*., 2011; Majumdar *et al*., 2012). Despite its important impact on the disposition of many other substrates, the effect of the endothelium on insulin activity in the liver has largely been overlooked (Fraser *et al*., 1995; Cogger *et al*., 2006; Cogger & Le Couteur, 2009). The main endothelial cells in the liver are the liver sinusoidal endothelial cells (LSECs), which are specialized endothelial cells that line the wall of the hepatic sinusoid. The thin cytoplasmic extensions of LSECs are perforated with fenestrations, which are nondiaphragmed, transcellular pores 50–150 nm in diameter. Between 2–20% of the surface of LSECs is covered by fenestrations clustered into groups called liver sieve plates (Wisse *et al*., 1985; Fraser *et al*., 1995; Cogger & Le Couteur, 2009). These ultrastructural features facilitate the transfer of plasma, solutes, and small particulate substrates between the blood and the hepatocytes. Under normal conditions, LSECs are a highly efficient ultrafiltration system; therefore, the influence of fenestrations on liver function is mostly seen in diseases and old age where their diameter and/or frequency are diminished (Cogger *et al*., 2006; Cogger & Le Couteur, 2009; Fraser *et al*., 2012). We propose that fenestrations also provide a portal for the hepatic uptake, and a subsequent clearance and activity of insulin, and that the loss of fenestrations, such as that occurs with old age, provides a novel mechanism for hepatic insulin resistance. Such a discovery would have a considerable clinical significance because it provides a new mechanism for insulin resistance seen in old age and many liver diseases where there is a reduction in fenestrations (Le Couteur *et al*., 2001; Cogger *et al*., 2006; Furrer *et al*., 2011).

Here, we have studied the effects of old age, together with the poloxamer 407 (P407) model of defenestration to examine the effects of loss of fenestrations on the disposition and activity of insulin in the liver and systemically. P407 is a nonionic surfactant that causes a marked defenestration of the LSECs within 24 h of administration without the development of fibrosis (Cogger *et al*., 2006; Warren *et al*., 2011). Defenestration seen in old age and induced in the acute setting by P407 has been shown to impair the transendothelial transfer and hepatic clearance of several substrates including lipoproteins, acetaminophen, and diazepam (Cogger *et al*., 2006; Mitchell *et al*., 2011, 2012). This study investigated the role of LSEC fenestrations in the hepatic disposition and action of insulin. Our results show that defenestration associated with old age and P407 interferes with the insulin transfer and uptake in the liver and impairs hepatic insulin signaling with systemic implications for glucose tolerance and insulin resistance. We show that defenestration is a mechanism for hepatic insulin resistance and hyperinsulinemia and specifically for the association between old age and insulin resistance.

## Results

### Old age is associated with an impaired hepatic and systemic disposition of insulin

**Figure 1:**
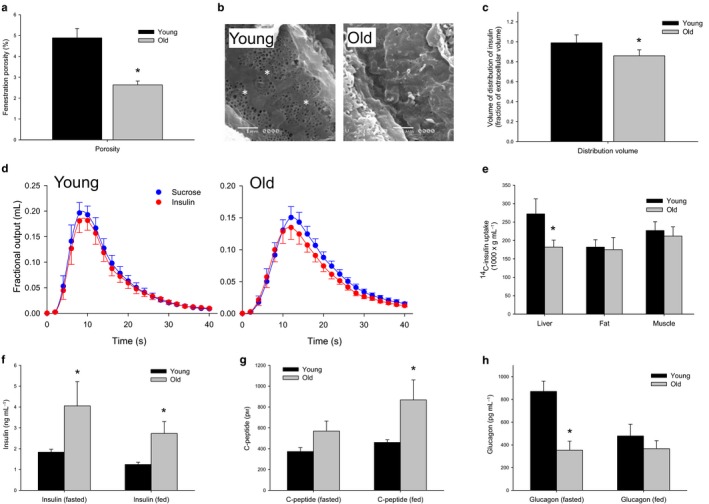
(a) Decreased porosity of the LSEC with age from scanning electron micrographs (*n *= 5 young and 5 old F344 rats *P* = 0.006). (b) Sample scanning electron micrographs of the LSEC to clearly illustrate the difference between young and defenestrated old rats, original micrographs taken at 15 000× magnification (fenestrations are indicated by an *). (c) There is a 20% reduction in the fractional volume of distribution of insulin with age (*n *= 9 young and 10 old F344 rats, *P* = 0.01) (d) MID outflow curves for insulin and the extracellular marker sucrose. Insulin exits the liver prior to sucrose in the old animals, indicating a restricted access to the entire extracellular space with age‐related defenestration. (e) ^14^C‐insulin uptake by the liver was found to be significantly reduced with age, but was found to be unchanged in the muscle and the fat (*n *= 10 young and 10 old mice, *P* < 0.05). (f) Fasting and fed insulin levels were found to be significantly elevated with age in C57Bl6 mice (*n *= 6 young and 5 old mice, *P* < 0.05). (g) C‐peptide levels were found to be significantly elevated with age in the fed state (*n *= 4 young and 5 old mice, *P* = 0.02) and (h) Glucagon levels were found to be suppressed in the fasting state in old mice (*n *= 6 young and 6 old mice, *P* = 0.02).

Hematoxylin and eosin staining of liver sections from the old and young male F344 rats confirmed that the animals were free of disease (Supplementary information). Scanning electron microscopy was performed to confirm age‐related defenestration of the LSEC that we and others have described previously in many species including rats, mice, and humans (Le Couteur *et al*., 2001, 2008). LSEC porosity was quantitated and showed a significant reduction in old F344 rats of about 50% compared to young rats (Fig. 1a–b).

To determine whether age‐related defenestration of the LSECs impairs the transendothelial transfer of insulin, we performed multiple indicator dilution (MID) studies in 3‐month‐old, 12‐ to 15‐month‐old, and 24‐ to 27‐month‐old rats using radiolabelled insulin and sucrose (Fig. 1c–d and Supplementary Information). Sucrose was used as the control tracer because it is a marker of the extracellular space and its transfer across the LSEC is unimpeded. Outflow insulin curves from the MID experiments are shown in Fig. 1d and Supplementary Information. In young rats, the volume of distribution of insulin was the same as that of the vascular and extracellular marker, sucrose, which indicates that insulin had access to the entire vascular and extracellular space. In old rats, the ratio of the volume of distribution to that of sucrose was about 80% and significantly less than in the young rats. This is consistent with the restriction of insulin to the vascular space (which is itself about three quarters of the extracellular space) in old age (Xu *et al*., 1990). In middle age, the ratio was intermediate between the young and old rats, consistent with the partial loss of fenestrations at this age (Le Couteur *et al*., 2001).

To determine whether the impaired transendothelial transfer of insulin impacted on the hepatic uptake of insulin, we measured the tissue uptake following an intravenous injection of ^14^C‐insulin. Insulin uptake by the liver was found to be reduced by about 30% in old C57Bl6 mice, but unchanged in the fat and the muscle (Fig. 1e). As a consequence of reduced hepatic uptake of insulin, the basal insulin concentrations were found to be significantly increased in the old mice both in the fasted and in the fed states when compared to their younger counterparts (Fig. 1f).

C‐peptide levels were found to be unchanged in the old mice during the fasted state (Fig. 1g), indicating that hyperinsulinemia is established through a reduced clearance of insulin by the liver rather than through an increased pancreatic insulin secretion. In the fed state, C‐peptide levels increased significantly with age, suggesting that an increased insulin secretion is required to maintain glucose tolerance with age. Glucagon levels were significantly lower in the old compared to the young mice when fasting (Fig. 1h).

### The effect of aging on systemic glucose tolerance and insulin resistance

**Figure 2:**
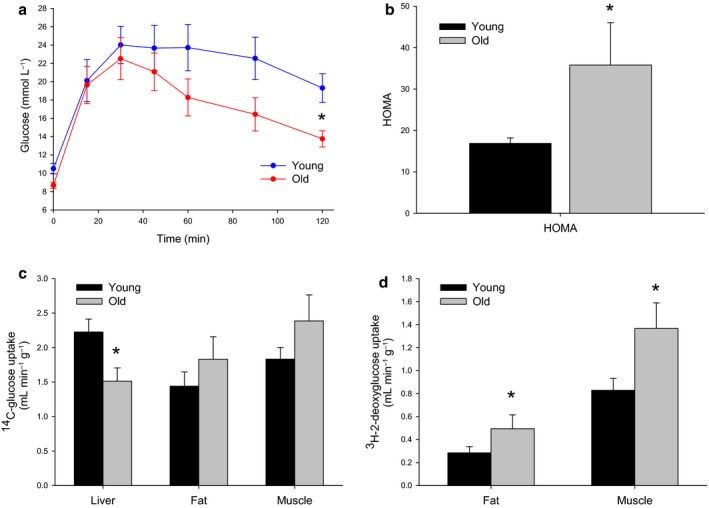
(a) Glucose tolerance was maintained despite hyperinsulinemia with old age in mice (*n *= 8 young and 5 old C57Bl6 mice). (b) The HOMA index was found to be significantly increased with age, reflecting the high insulin levels (*n *= 6 young and 5 old mice, *P* = 0.03). (c) There was a significant reduction in hepatic uptake of ^14^C‐glucose and a trend toward increased uptake by the muscle and the fat with age (*n *= 8 young and 5 old mice, *P* < 0.05). (d) ^3^H‐2‐deoxyglucose incorporation into the fat and the muscle was found to be significantly increased with age (*n *= 8 young and 5 old mice, *P* < 0.05).

Although the old mice were hyperinsulinemic, the glucose tolerance test was not impaired, in fact tended to be lower (Fig. 2a). However, because of the very high insulin levels, the homeostatic model assessment index for insulin resistance (HOMA‐IR), which is calculated from the product of the fasting glucose and insulin levels, was found to be increased by more than twofold in the old mice, indicating the insulin resistance (Fig. 2b).

To determine which tissues were contributing to the insulin resistance, ^14^C‐glucose and ^3^H‐2‐deoxyglucose were administered to the mice during glucose tolerance tests and the uptake was measured in the muscle, the white adipose tissue (WAT), and the liver. The uptake of ^14^C‐glucose was found to be significantly reduced in the liver, but this was associated with an increase in muscle uptake (Fig. 2c). In addition, there was a significant increase in ^3^H‐2‐deoxyglucose uptake, which is not a substrate for liver uptake and metabolism, in both muscle and fat with age (Fig. 2d). The data indicate that the reduction in hepatic glucose uptake and hyperinsulinemia are associated with a compensatory age‐related increase in glucose uptake in the muscle and the fat (as well as depleted glycogen stores), thereby normalizing the glucose tolerance test.

### The effect of aging on hepatic insulin activity

**Figure 3:**
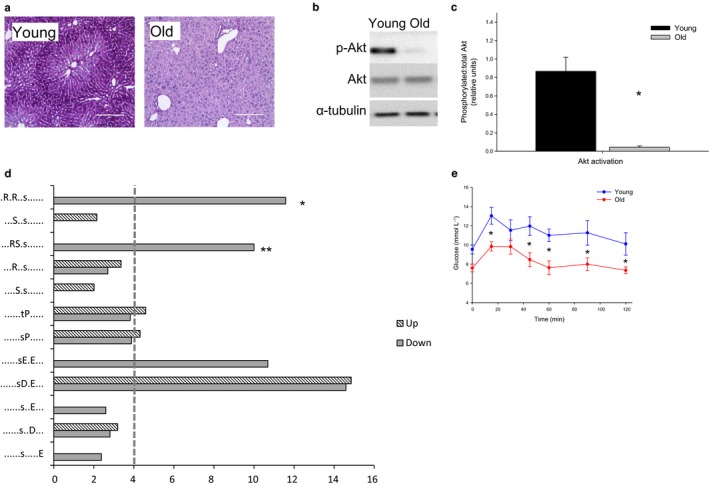
(a) PAS staining of the liver in young and old rats showing a significantly reduced glycogen storage with age (*n *= 8 young and 5 old mice). (b) Western blots of Akt and pAkt showing a significantly decreased phosphorylation in insulin‐stimulated old mouse livers compared to young. (c) Densitometry for AKT:pAkt (*n *= 6 young and 6 old mice, *P* = 0.002). (d) Fold overrepresentation of kinase recognition sequences from phosphoproteome analysis of young versus aged livers. Reduced Akt signaling is indicated by the prevalence of Akt recognition sequences * = ‘R‐x‐R‐x‐x‐pS’ (11.5‐fold) and ** = ‘R‐S‐x‐pS’ (10‐fold) in the set of phosphopeptides with a reduced abundance in aged livers. Dotted line indicates a cutoff for significant fold change (>4‐fold). (e) Pyruvate tolerance tests revealed a decreased gluconeogenesis with age (*n *= 8 young and 8 old mice, *P* = 0.003).

Consistent with the selective impact of age‐related defenestration on hepatic insulin sensitivity, hepatic glycogen storage measured by PAS staining showed a marked reduction in glycogen in the old mice (Fig. 3a). Phosphorylation of the hepatocellular insulin receptor Akt was found to be reduced in the old mice (Fig. 3b–c). To further probe this, we utilized a large‐scale, unbiased phosphoproteomic approach and liquid chromatography coupled to tandem mass spectrometry (LC‐MS/MS) to identify the changes in protein and phosphopeptide abundance, which enabled a signal pathway mapping in the liver tissues from the young and old mice. LC‐MS/MS of phosphopeptide‐enriched samples identified 7208 sites of phosphorylation (*n *= 5156 phosphopeptides from *n *=* *2400 proteins), of which 1580 were found to be statistically significantly altered in abundance (*z*‐score <−1.00 or >+1.00; Supplementary data). Nonphosphorylated peptides from the same samples were also identified and revealed only 281 proteins (*z*‐score <−1.96 or >+1.96; Supplementary data) that were found to be significantly altered in abundance, confirming that the major changes at the biochemical level between the young and old mouse livers were those associated with signaling. We next specifically examined the set of altered phosphopeptides and used functional cluster analysis to identify Kyoto Encyclopaedia of Genes and Genomes (KEGG) pathways associated with aging in the liver. Functional clusters contained within this dataset were compared against the mouse genome to determine their overrepresentation compared to background. The most overrepresented KEGG pathways were ErbB (*p*‐value 9.77 e^−9^), neurotrophin (4.10 e^−7^), GnRH (4.27 e^−7^), MAPK (7.96 e^−7^), and insulin (1.05 e^−6^) signaling. The diversity of these pathways reflects the likely multifactorial nature of aging; however, the changes associated with insulin signaling are consistent with the reduced access of insulin to hepatocytes in old age. We next performed a site‐specific analysis of the phosphopeptides that were found to be statistically significantly altered in aged livers by performing kinase recognition motif analysis using MotifX. These data showed that the Akt recognition motifs R‐X‐R‐X‐X‐pS and R‐S‐X‐pS were found to be enriched 11.5‐fold and 10‐fold, respectively, compared with background in the dataset of peptides displaying a reduced phosphorylation with aging (Fig. 3d), which is consistent with the Akt Western blot data and confirms a significant reduction in Akt signaling in aged mouse livers. Motifs containing acidic residues, consistent with casein kinase 2 (CK2) activation, were also found to be enriched; however, these were found in both the up‐ and downregulated phosphopeptide datasets.

To identify the gene expression that might be altered by aging, a qRT–PCR screening array for 84 key genes in the insulin signaling pathway was conducted (Supplementary information). Gene expression profiles showed no changes in insulin signaling pathway genes, which was consistent with the proteomic data and again confirmed that changes in phosphorylation‐mediated signaling, rather than expression, were associated with aging. Fatty acid synthase, a protein responsible for fatty acid synthesis, was the only gene in this panel shown to be downregulated with age in the liver.

We also performed a pyruvate tolerance test in mice (Fig. 3e) to examine the impact of age on the insulin‐mediated inhibition of gluconeogenesis. Old age was associated with a lower conversion of pyruvate to glucose. This is consistent with the observation that in rodents some of the effects of insulin on hepatic gluconeogenesis are mediated via the brain, rather than a direct effect on hepatocytes (Rojas & Schwartz, 2014). In this situation, age‐related hyperinsulinemia will drive brain‐mediated inhibition of gluconeogenesis even though its direct hepatic activity is reduced.

### The P407 model of acute defenestration confirms the effects of LSEC fenestrations on hepatic and systemic disposition of insulin

**Figure 4:**
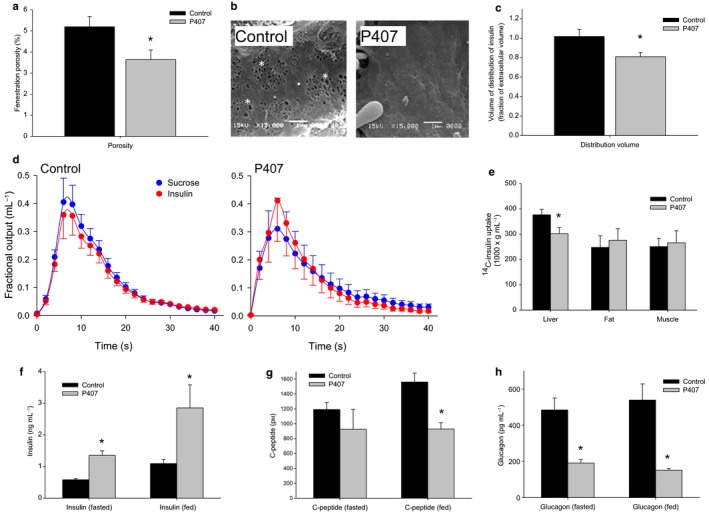
(a) Acute defenestration of the LSEC was induced by P407 within 24 h of injection as measured from scanning electron micrographs (*n *= 10 control and 11 P407‐treated rats, *P* < 0.05). (b) Sample scanning electron micrographs of the LSEC to illustrate more pronounced examples of defenestration in control and P407‐treated rats (original micrographs 15 000× magnification, fenestrations are indicated by an *). (c) There was a 20% reduction in the fractional volume of distribution of insulin with P407‐induced defenestration (*n *= 12 control and 8 P407‐treated rats, *P* = 0.01). (d) MID outflow curves for insulin and the extracellular marker, sucrose. As seen with the age‐related defenestration, insulin exits the liver prior to sucrose in the P407‐treated rats, indicating a restricted access to the entire extracellular space. (e) ^14^C‐insulin uptake by the liver was found to be significantly reduced with defenestration, but was found to be unchanged in the muscle and the fat (*n *= 6 control and 6 P407‐treated rats *P* = 0.04). (f) Fasting and fed insulin levels were found to be significantly elevated following the P407‐induced defenestration (*n *= 6 control and 6 P407‐treated rats, fasting *P* < 0.05, fed *P* = 0.005). (g) C‐peptide levels were found to be significantly decreased with P407‐induced defenestration in the fed state (*n *= 5 control and 5 P407‐ rats, *P* = 0.03). (h) Glucagon levels were found to be suppressed in the fasting and fed states in P407‐treated rats (*n *= 4 control and 4 P407‐treated rats, fasting *P* = 0.02; fed *P* = 0.016).

Because aging is a multifactorial process where many fundamental cellular processes are affected, we further investigated the role of the LSEC in hepatic insulin handling using an acute model of defenestration generated by P407. Hematoxylin and eosin staining of the liver sections from the control and P407 rats confirmed that they were free of diseases (Supplementary information). P407‐induced defenestration was confirmed with scanning electron microscopy, and the significant reduction in LSEC porosity of about 30% was similar to that seen with aging (Fig. 4a–b). We also confirmed that P407 was associated with the increased blood levels of cholesterol and triglycerides (Supplementary information), which is a well‐recognized effect of this agent (Johnston, 2004; Cogger *et al*., 2006).

P407‐induced defenestration was associated with a significant reduction in the hepatic volume of distribution of insulin as a fraction of the extracellular sucrose volume of about 20% (Fig. 4c–d). To determine whether this reduction in the transendothelial transfer of insulin impacted on the hepatic uptake of insulin, we measured the tissue uptake following an intravenous injection of ^14^C‐insulin. Insulin uptake by the liver was found to be reduced by 20% in P407‐treated animals (Fig. 4e), but was found to be unchanged in the fat and the muscle. As a consequence, insulin levels were significantly higher in P407 rats as compared to control in both fed and fasted states (Fig. 4f).

C‐peptide levels were significantly lower in the treatment group during fed state (Fig. 4g), showing an acute suppression of pancreatic insulin secretion in response to the reduced clearance of insulin by the liver. Glucagon levels in the fasting and fed states were found to be significantly reduced in the P407 animals as seen with old mice (Fig. 4h).

### The effect of P407 on systemic glucose tolerance and insulin resistance

**Figure 5:**
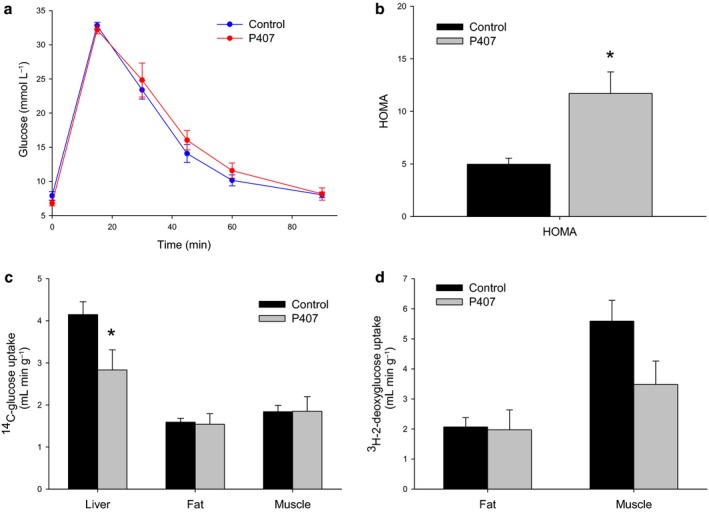
(a) Glucose tolerance was found to be unchanged by P407‐induced defenestration (*n *= 10 control and 8 P407‐treated rats). (b) The HOMA index was found to be significantly increased by P407‐induced defenestration (*n *= 6 control and 6 P407‐treated rats, *P* = 0.002). (c) There was a significant reduction in hepatic uptake of ^14^C‐glucose with P407, but no change in muscle and fat uptake (*n *= 10 control and 8 P407‐treated rats, *P* < 0.05). (d) ^3^H‐2‐deoxyglucose incorporation into the fat and the muscle was found to be unchanged following P407 treatment, although there was a trend toward the reduced muscle uptake (*n *= 10 control and 8 P407‐treated rats, *P* = 0.06).

The glucose tolerance test was found to be not significantly influenced by P407 (Fig. 5a). However, because of the elevated insulin levels, the HOMA‐IR was found to be increased by more than twofold in the P407 rats (Fig. 5b). In addition, the uptake of ^14^C‐glucose was found to be reduced in the liver, but not the muscle and the fat (Fig. 5c). There was no significant change in the uptake of ^3^H‐2‐deoxyglucose into the muscle and the fat following P407 treatment (Fig. 5d), although the reduction in the muscle uptake was pronounced (*P* = 0.06). These data suggest that the liver is the primary target for the effects of P407 on glucose handling.

### The effect of P407 on hepatic insulin activity

**Figure 6:**
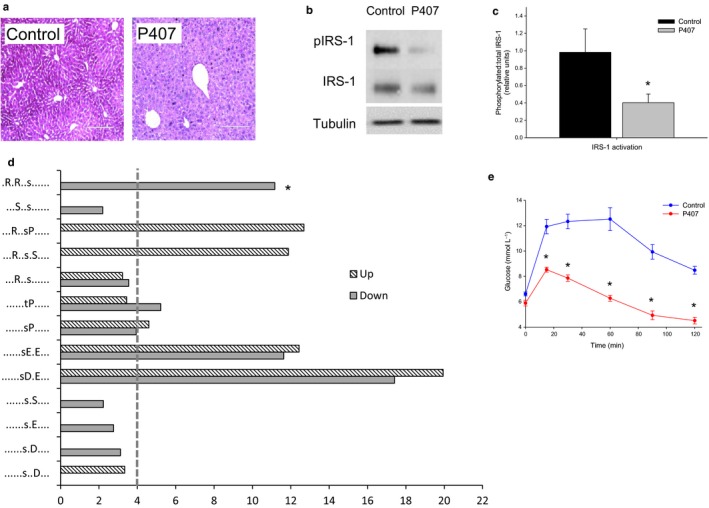
(a) PAS staining of the liver in control and P407‐treated rats showing a significantly reduced glycogen storage with defenestration (*n *= 10 control and 8 P407‐treated rats). (b) In the setting of insulin stimulation, Western blots of IRS‐1 showed a reduced phosphorylation in the setting of P407‐induced defenestration. (c) Quantitation of IRS‐1 phosphorylation in P407‐induced defenestration showed a reduction of approximately 60% (*n *= 9 control and 9 P407‐treated rats, *P* < 0.05). (d) Fold overrepresentation of kinase recognition sequences from phosphoproteome analysis of control versus P407‐treated livers. Reduced Akt signaling is indicated by the prevalence of Akt recognition sequence *= ‘R‐x‐R‐x‐x‐pS’ (11‐fold) in the set of phosphopeptides with the reduced abundance in P407‐treated livers. Dotted line indicates a cutoff for significant fold change (>4‐fold) (e) As seen with aging, pyruvate tolerance tests revealed an impaired gluconeogenesis (*n *= 13 control and 9 P407‐treated rats, *P* < 0.05).

Hepatic glycogen storage measured by PAS staining was found to be markedly reduced following P407 treatment, which is consistent with a diminished insulin action (Fig. 6a). Western blot analysis revealed that phosphorylation of IRS‐1, the phosphorylation target of the activated insulin receptor, was found to be significantly reduced by over 50% in P407 rats as compared to controls (Fig. 6b–c). Next, we again performed large‐scale phosphoproteomics to determine the effects of P407 on hepatic cell signaling pathways to further delineate the effect of fenestration loss in the absence of the multifactorial processes involved in aging. LC‐MS/MS identified 1,480 sites of phosphorylation that were found to be statistically significantly altered in abundance (*z*‐score <−1.00 or > +1.00; Supplementary information). Analysis of nonphosphorylated peptides revealed only 189 proteins that were found to be significantly altered in abundance, and these contained no discernible functional or spatial pattern. Proteins containing significantly altered phosphopeptides were subjected to functional cluster analysis and compared against the mouse genome as background. The most overrepresented KEGG functional pathway was the insulin signaling pathway (*p*‐value 9.24 e^−10^). Site‐specific analysis using MotifX again showed a 11‐fold overrepresentation of the Akt recognition sequence (R‐X‐R‐X‐X‐pS) in the set of phosphopeptides significantly reduced by P407 treatment (Fig. 6d), which is consistent with the data observed for aged liver tissue.

We performed a qRT–PCR screening array for key genes in the rat insulin signaling pathways (Supplementary information). Gene expression profiles showed that IRS‐2 was found to be upregulated (1.76‐fold; *P *=* *0.02) and thyroglobulin, which has a role as secondary effector target genes for insulin signaling, was found to be downregulated (1.88‐fold; *P *=* *0.01, Supplementary information). There were also upregulation of leptin‐coding gene Cebpa that is a primary target gene for insulin signaling (6.08‐fold) and downregulation of G6pc gene involved in glucose and glycogen metabolism (3.53‐fold), but this was not significant.

We measured the pyruvate tolerance test (Fig. 6e) to examine the impact of P407 on insulin‐mediated inhibition of gluconeogenesis. As we found with old age, P407 was associated with a lower conversion of pyruvate to glucose, presumably an effect of hyperinsulinemia on brain‐mediated inhibition of gluconeogenesis.

## Discussion

We have demonstrated that defenestration of the LSEC during aging and after the treatment with P407 restricts the insulin access to the insulin receptor on the hepatocyte membrane through an impaired transendothelial transfer. This results in hyperinsulinemia, impaired hepatic insulin signaling, depleted glycogen stores, compensatory increases in the muscle and fat uptake of insulin and glucose, and dysregulated gluconeogenesis. In the fat and the muscle, the blood vessels are the rate‐limiting step for the uptake of insulin (Sandqvist *et al*., 2011; Majumdar *et al*., 2012). Our data show that under physiological conditions, the liver endothelium does not provide a barrier for the uptake of insulin, which attests to its remarkable role in facilitating the transfer of substrates between the blood and the liver cells (Le Couteur *et al*., 2005). However, in conditions associated with the structural changes in the LSEC, the liver blood vessels become a barrier for the transfer of insulin. This is a significant development in our understanding of insulin resistance seen with age and liver disease.

As we have previously reported, aging and P407 treatment were associated with a significant defenestration of the LSEC (Cogger *et al*., 2006; Mitchell *et al*., 2011; Warren *et al*., 2011). Using multiple indicator dilution methodology in rats, we determined the disposition of insulin in perfused livers and showed that aging and P407‐induced defenestration were associated with a restricted access of insulin to the extravascular space. We have previously shown that defenestration associated with old age and P407 is mechanistically linked to the reduced transendothelial transfer of other substrates including lipoproteins (Hilmer *et al*., 2005; Cogger *et al*., 2006) and both albumin‐bound and dissolved medications (Le Couteur *et al*., 2005; Mitchell *et al*., 2011). Fenestration loss is also known to occur in streptozotocin‐induced diabetic baboons and rats (Jamieson *et al*., 2007; McMahon *et al*., 2013), which might further contribute to diabetes in these models.

Accompanying an impaired transendothelial access of insulin to the hepatocytes, there were significant systemic changes including reduced hepatic insulin uptake and clearance, increased insulin levels and insulin resistance. This was associated with insulin resistance as assessed by the HOMA, but the glucose tolerance test was normal in the aged animals. We propose that this is secondary to long‐term age‐related compensatory effects on glucose uptake in the muscle and the fat, and the reduction in hepatic glycogen storage.

Despite elevated circulating insulin levels, aging and P407 treatment were associated with the impairment of hepatic insulin signaling as measured by a full hepatic phosphoproteome screen using LC‐MS/MS and glycogen stores. Insulin signaling was found to be affected by both aging and P407. Western blot showed a reduced hepatic IRS‐1 phosphorylation in insulin‐stimulated livers from P407‐treated animals. This was associated with a compensatory increase in the expression of IRS‐2. A similar finding was observed in the insulin‐stimulated liver from LIRKO mice, where there was a fivefold increase in IRS‐2 expression in the liver due to the loss of insulin signaling (Michael *et al*., 2000). Additionally, large‐scale data confirmed the reduced insulin signaling in both aged and P407‐treated livers on SHC‐1, a second phosphorylation target of the insulin receptor.

In summary, patent fenestrations are required for hepatic insulin uptake, clearance, and signaling. We acknowledge that a limitation of this study is the use of two species, mice and rats, which have quantitatively different glucose metabolism. Even so, the data suggest that defenestration of the LSEC with old age provides a new mechanism that might contribute to insulin resistance along with other mechanisms such as age‐related changes in insulin sensitivity in muscle. Maintaining the structural integrity of the LSEC is a potential therapeutic target for insulin resistance in old age.

## Experimental procedures

### Animals

For the aging studies, male Fischer 344 rats and male C57/Bl6 mice aged 3 months, 12‐15 months, and 24–27 months were obtained from the National Institutes of Aging (Baltimore, MD). The animals were allowed free access to water and standard chow. Rats were used for the multiple indicator dilution studies because these studies are only technically feasible in larger livers. Mice were used for the remaining *in vivo* studies because of the limited availability of old rats.

For the P407 studies, male Fischer 344 rats weighing 200 g were obtained from Animal Research Centre (Perth, Australia). The animals were allowed free access to water and standard chow. The treatment group received an intraperitoneal injection (i.p.) of P407 (1 g/kg; BASF Ltd, Southbank, Vic., Australia). Control animals included those that received a volume‐matched i.p. injection of normal saline or were untreated. There were no differences between control animals given saline or no treatment; therefore, their data were pooled.

All animals were treated in accordance with Animal Care guidelines. This study was approved by the Sydney Local Health District Animal Welfare Committee (AWC Protocol #2012/005A) and the National Institute on Aging Animal Care and Use Committee (Protocol number: 429‐TGB‐2017).

### Liver perfusion

Liver perfusions and MID experiments were performed in 3‐month‐old, 24‐month‐old, young controls, and young P407 rats as described previously (Cogger *et al*., 2006; Mitchell *et al*., 2011, 2012). For MID experiments, ^3^H‐sucrose and ^14^C‐insulin were used to spike the injectate. Analysis for ^14^C‐ and ^3^H‐specific activity from outflow samples was performed in a Packard 1600TR liquid scintillation counter, and data were used to generate dose‐normalized outflow curves. The area under the curve (AUC), area under the moment of the curve (AUMC), mean transit time (MTT), and the recovery of each indicator were determined.

### Blood lipid analysis

Prior to cannulation, the blood samples were taken from the inferior vena cava for lipid analysis. Total cholesterol and triglyceride levels were determined using 917 autoanalyzer (Roche Pty Ltd, Basel Switzerland) by the Concord Hospital NATA‐accredited Biochemistry laboratories.

### Scanning electron microscopy

At the completion of the multiple indicator dilution experiments, the livers were perfusion‐fixed with 3% glutaraldehyde/2% paraformaldehyde in 0.1 m sodium cacodylate buffer as described previously (Cogger *et al*., 2006; Mitchell *et al*., 2012). Fenestrations in the liver endothelium were examined using a Jeol 6380 scanning electron microscope at 15 000× magnification. Ten random images per sample were taken for the analysis of fenestrations diameter and porosity using ImageJ software.

### Glucose tolerance, tracer analysis, and pyruvate tolerance tests

Animals were fasted for 6 h, followed by the administration of glucose (2 g kg^−1^ i.v.) spiked with 10 μCi ^14^C‐glucose for the assessment of insulin action in the liver, the fat, and the muscle and 10 μCi ^3^H‐2‐deoxyglucose for the assessment of glucose uptake in the fat and the muscle. After 15, 30, 45, 60, and 90 min, glucose levels were measured with a handheld glucose meter (Accu‐Check Performa, Roche, Sydney, NSW, Australia). At 90 min, the animals were sacrificed and the liver, the adipose tissue, and the quadriceps muscle were excised, weighed, and either snap‐frozen in liquid nitrogen or placed in 4% phosphate‐buffered paraformaldehyde, dehydrated, and embedded in paraffin for histology. Blood was collected at 0 and 90 min to determine the insulin, C‐peptide, and glucagon levels (Rat Ultrasensitive Insulin ELISA & Rat C‐peptide ELISA: Alpco Diagnostics Caringbah, NSW, Australia; Glucagon EIA kit: Sigma Aldrich, Castle Hill, NSW, Australia) and for the calculation of HOMA index (the product of the fasting glucose and insulin levels) in the fasted and fed states. Blood samples were deproteinized using BaOH and ZnSO_4_ to quantitate plasma radioactivity by liquid scintillation counting. Incorporation of radiolabelled glucose to glycogen and ^3^H‐2‐deoxyglucose uptake were determined according to the methods as described previously (Fueger *et al*., 2003; Cooney *et al*., 2004). The uptake of insulin into the liver, the fat, and the muscle was performed in another group of rats, where ^14^C‐insulin (1 μCi g^−1^) was injected via the inferior vena cava of anesthetized animals. Five minutes later, the animals were exsanguinated by cardiac puncture and the blood, the liver, the white adipose tissue (WAT), and the quadriceps muscle tissue were collected for the measurement of ^14^C‐insulin (Sodoyez *et al*., 1980; Philippe *et al*., 1981). For pyruvate tolerance tests, the animals were fasted for 6 h prior to an i.p injection of pyruvate (2 g kg^−1^; Sigma Aldrich, Castle Hill, Australia) and glucose levels were read using a handheld glucose meter (Accu‐Check Performa, Roche, Australia) through tail bleeds at 0, 15, 30, 60, and 90 min. Total AUC was calculated using the trapezoidal formula.

### Hematoxylin and eosin and periodic acid–schiff staining

Paraffin‐embedded liver specimens were cut at 4 μm, mounted on glass slides, then subjected to hematoxylin and eosin staining for the assessment for diseases and periodic acid–Schiff (PAS) staining for glycogen (Gahrton, 1964) and examined with light microscopy at 40× magnification.

### Phosphoproteomic studies

Liver tissue was solubilized in SDS–Tris buffer in the presence of phosphatase and kinase inhibitors prior to protein precipitation. Following tryptic digestion, 250 μg of peptide was labeled with one of four isobaric tags (iTRAQ, Sciex) prior to phosphoproteomic enrichment (Jensen *et al*., 2009; Engholm‐Keller *et al*., 2012). This enrichment generated 3 peptide populations, singly and multiply phosphorylated peptides (11 fractions) and nonmodified peptides (16 fractions). Identification and quantitation of phosphorylated and nonphosphorylated peptides was performed on an Orbitrap Velos Pro mass spectrometer in data‐dependent acquisition (DDA) mode. All experiments were performed in duplicate. Data were analyzed using Proteome Discoverer (version 1.4; Thermo Scoresby, VIC, Australia) and searched using an in‐house MASCOT server against the UniProt *Mus musculus* database with the following parameters: 2 missed cleavages, 20 ppm mass error (MS), and 0.2 Da mass error (MS/MS); iTRAQ searched as a static modification: carbamidomethyl (Cys), oxidation (Met), acetylation (protein N‐term), and cyclization (Glu and Asp) as dynamic modifications. A false discovery rate of 0.05 was applied to phosphopeptides, with a stricter 0.01 FDR applied to the nonmodified cohort. For the analysis of MS data, normalization of iTRAQ reporter ions was calculated using the sum of all intensities approach combining both phosphorylated and nonphosphorylated peptide spectral matches (PSM), prior to ratio calculation using either young or control as the denominator for old and P407‐treated animals, respectively. Log2 ratios and *z*‐scores were calculated for each PSM, with *z*‐scores determined using a sliding scale based on MS signal intensity (a measure of MS/MS data quality). Median *z*‐scores were calculated with weighted average used to compare medians across experiments for each phosphosite (phosphoproteome enriched), peptide and protein (nonphosphorylated only). To be deemed significantly altered, median *z*‐scores were required to be >1.00 or <−1.00 for phosphosites. Median *z*‐scores for each nonphosphorylated peptide were calculated preceding the determination of protein *z*‐scores. For a protein to be deemed significantly altered, median *z*‐scores >1.96 or <−1.96, equivalent to *P* values of <0.05, were required. Bioinformatic analysis was performed to reveal the pathways associated with aging or P407 treatment using STRING (Jensen *et al*., 2009; Szklarczyk *et al*., 2011; Franceschini *et al*., 2013) and KEGG (Kanehisa & Goto, 2000; Kanehisa *et al*., 2014) pathway analysis. Site‐specific phosphopeptide analyses were conducted using MotifX (http://motif-x.med.harvard.edu/motif-x.html) to identify fold overrepresentation of kinase recognition sequences, and PhosphoSite Plus to identify insulin‐regulated phosphorylation sites in proteins represented in the KEGG insulin signaling pathway.

Frozen liver tissue samples were homogenized (Qiagen TissueLyser) in RIPA buffer containing Tris–HCl, NaCl, Triton X‐100, Na‐deoxycholate, SDS, and Roche protease inhibitor tablets (Complete, EDTA‐free Protease Inhibitor Cocktail). Protein concentrations were determined using the BCA method (Thermo Scientific Pierce BCA Protein Assay Kit). The protein extracts were subjected to immunoblotting according to the protocol as described previously (Hoehn *et al*., 2008). Antibodies against the following proteins were used: p‐IRS‐1 (Tyr895), IRS‐1, p‐AKT (Ser473), AKT, IRS‐2, and α‐tubulin (Cell Signaling Technology, Arundel, Qld, Australia).

### Reverse–transcription and quantitative real‐time PCR array analysis

Total RNA was isolated from the frozen liver tissue samples using RNeasy Plus Mini Kit (Qiagen Pty Ltd, Chadstone, Vic., Australia) according to the manufacturer's instructions, with DNase treatment included; 1 μg of total RNA was reverse‐transcribed using RT^2^ First strand kit. The cDNA was then added to the RT^2^ SYBR green qPCR mastermix and loaded onto 96‐well RT^2^ Profiler PCR array plate (PARN‐030‐ZD; Qiagen Pty Ltd) and amplified on Bio‐Rad CFX Connect Real Time PCR Detection system for 40 cycles. PCR array data were analyzed according to the manufacturer's instructions. Relative gene expression was determined using the ∆∆C_t_ method normalized against five housekeeping genes, and the changes in gene expression were shown as a fold increase or decrease compared to control group.

### Statistical analyses

All values are expressed as the mean ± SEM. Statistical significance was calculated using two‐tailed Student's t‐test using Sigmaplot (Systat Software, Erkrath, Germany, v11.0).

## Funding

We acknowledge the financial support from the Ageing and Alzheimer's Research Foundation. This study was funded in part by the National Institute on Aging, National Institutes of Health, and facilitated by the Sydney Mass Spectrometry Core Facility (MSCF).

## Conflict of interest

None declared.

## Author contributions

DLC, VC, SJS, SNH, RdC, and DAS conceptualized the study. VC, DLC, and MM planned the study. MM and VC led the majority of experiments, while LW, AD, SYCL, and DAS performed Western blot analysis; SJC and MYW performed proteomics; and SJM, DB, DN, SMS, JM, and SMS assisted with the aging studies under the supervision of RdC. All authors contributed to the analysis of the data. VC, MM, SJC, and DLC wrote the first draft.

## Supporting information


**Fig. S1** H&E Staining of liver tissue from the young and old rats and control and P07‐treated rats.
**Fig. S2** Insulin and sucrose curves and volume of distribution of insulin for the liver of middle aged F344 rats.
**Fig. S3** Supplementary information for phosphoproteomics.
**Fig. S4** RT–PCR data for Insulin signaling pathway in young versus old livers and control versus P407 livers.
**Fig. S5** Increased blood levels of cholesterol and triglycerides following P407 treatment.

## References

[acel12481-bib-0001] Bluher M , Michael MD , Peroni OD , Ueki K , Carter N , Kahn BB , Kahn CR (2002) Adipose tissue selective insulin receptor knockout protects against obesity and obesity‐related glucose intolerance. Dev. Cell 3, 25–38. 12110165 10.1016/s1534-5807(02)00199-5

[acel12481-bib-0002] Bose SK , Ray R (2014) Hepatitis C virus infection and insulin resistance. World J. Diabetes 5, 52–58. 24567801 10.4239/wjd.v5.i1.52 PMC3932427

[acel12481-bib-0003] Bruning JC , Michael MD , Winnay JN , Hayashi T , Horsch D , Accili D , Goodyear LJ , Kahn CR (1998) A muscle‐specific insulin receptor knockout exhibits features of the metabolic syndrome of NIDDM without altering glucose tolerance. Mol. Cell 2, 559–569. 9844629 10.1016/s1097-2765(00)80155-0

[acel12481-bib-0004] Chai SY , Pan XY , Song KX , Huang YY , Li F , Cheng XY , Qu S (2014) Differential patterns of insulin secretion and sensitivity in patients with type 2 diabetes mellitus and nonalcoholic fatty liver disease versus patients with type 2 diabetes mellitus alone. Lipids Health Dis. 13, 7. 24397589 10.1186/1476-511X-13-7 PMC3896963

[acel12481-bib-0005] Cogger VC , Le Couteur DG (2009) Fenestrations in the liver sinusoidal endothelial cell In The Liver: Biology and Pathobiology (Arias IM, Wolkoff A, Boyer JL, Shafritz DA, Fausto N, Alter H, Cohen A, eds). Hoboken, NJ: John Wiley & Sons, Ltd, pp. 387–404.

[acel12481-bib-0006] Cogger VC , Hilmer SN , Sullivan D , Muller M , Fraser R , Le Couteur DG (2006) Hyperlipidemia and surfactants: the liver sieve is a link. Atherosclerosis 189, 273–281. 16458315 10.1016/j.atherosclerosis.2005.12.025

[acel12481-bib-0007] Cooney GJ , Lyons RJ , Crew AJ , Jensen TE , Molero JC , Mitchell CJ , Biden TJ , Ormandy CJ , James DE , Daly RJ (2004) Improved glucose homeostasis and enhanced insulin signalling in Grb14‐deficient mice. EMBO J. 23, 582–593. 14749734 10.1038/sj.emboj.7600082 PMC1271812

[acel12481-bib-0008] Engholm‐Keller K , Birck P , Storling J , Pociot F , Mandrup‐Poulsen T , Larsen MR (2012) TiSH–a robust and sensitive global phosphoproteomics strategy employing a combination of TiO2, SIMAC, and HILIC. J. Proteomics. 75, 5749–5761. 22906719 10.1016/j.jprot.2012.08.007

[acel12481-bib-0009] Fink RI , Kolterman OG , Griffin J , Olefsky JM (1983) Mechanisms of insulin resistance in aging. J. Clin. Investig. 71, 1523–1535. 6345584 10.1172/JCI110908 PMC370358

[acel12481-bib-0010] Fisher SJ , Kahn CR (2003) Insulin signaling is required for insulin's direct and indirect action on hepatic glucose production. J. Clin. Investig. 111, 463–468. 12588884 10.1172/JCI16426 PMC151923

[acel12481-bib-0011] Franceschini A , Szklarczyk D , Frankild S , Kuhn M , Simonovic M , Roth A , Lin J , Minguez P , Bork P , von Mering C , Jensen LJ (2013) STRING v9.1: protein‐protein interaction networks, with increased coverage and integration. Nucleic Acids Res. 41, D808–D815. 23203871 10.1093/nar/gks1094 PMC3531103

[acel12481-bib-0012] Fraser R , Dobbs BR , Rogers GW (1995) Lipoproteins and the liver sieve: the role of fenestrated sinusoidal endothelium in lipoprotein metabolism, atherosclerosis, and cirrhosis. Hepatology 21, 863–874. 7875685

[acel12481-bib-0013] Fraser R , Cogger VC , Dobbs B , Jamieson HA , Warren A , Hilmer SN , Le Couteur DG (2012) The liver sieve and atherosclerosis. Pathology 44, 181–186. 22406487 10.1097/PAT.0b013e328351bcc8

[acel12481-bib-0014] Fueger PT , Heikkinen S , Bracy DP , Malabanan CM , Pencek RR , Laakso M , Wasserman DH (2003) Hexokinase II partial knockout impairs exercise‐stimulated glucose uptake in oxidative muscles of mice. Am. J. Physiol. Endocrinol. Metab. 285, E958–E963. 12865258 10.1152/ajpendo.00190.2003

[acel12481-bib-0015] Furrer K , Rickenbacher A , Tian Y , Jochum W , Bittermann AG , Kach A , Humar B , Graf R , Moritz W , Clavien PA (2011) Serotonin reverts age‐related capillarization and failure of regeneration in the liver through a VEGF‐dependent pathway. Proc. Natl Acad. Sci. USA 108, 2945–2950. 21282654 10.1073/pnas.1012531108 PMC3041135

[acel12481-bib-0016] Gahrton G (1964) Microspectrophotometric quantitation of the Periodic Acid‐Schiff (PAS) reaction in human neutrophil leukocytes based on a model system of glycogen microdroplets. Exp. Cell Res. 34, 488–506. 14170005 10.1016/0014-4827(64)90234-4

[acel12481-bib-0017] Hilmer SN , Cogger VC , Fraser R , McLean AJ , Sullivan D , Le Couteur DG (2005) Age‐related changes in the hepatic sinusoidal endothelium impede lipoprotein transfer in the rat. Hepatology 42, 1349–1354. 16317689 10.1002/hep.20937

[acel12481-bib-0018] Hoehn KL , Hohnen‐Behrens C , Cederberg A , Wu LE , Turner N , Yuasa T , Ebina Y , James DE (2008) IRS1‐independent defects define major nodes of insulin resistance. Cell Metab. 7, 421–433. 18460333 10.1016/j.cmet.2008.04.005 PMC2443409

[acel12481-bib-0019] Izzo JL , Bartlett JW (1969) Insulin‐glucose dispersion and interaction system. Liver control mechanisms. Arch. Intern. Med. 123, 272–283. 4886257

[acel12481-bib-0020] Jamieson HA , Cogger VC , Twigg SM , McLennan SV , Warren A , Cheluvappa R , Hilmer SN , Fraser R , de Cabo R , Le Couteur DG (2007) Alterations in liver sinusoidal endothelium in a baboon model of type 1 diabetes. Diabetologia 50, 1969–1976. 17604976 10.1007/s00125-007-0739-4

[acel12481-bib-0021] Jensen LJ , Kuhn M , Stark M , Chaffron S , Creevey C , Muller J , Doerks T , Julien P , Roth A , Simonovic M , Bork P , von Mering C (2009) STRING 8–a global view on proteins and their functional interactions in 630 organisms. Nucleic Acids Res. 37, D412–D416. 18940858 10.1093/nar/gkn760 PMC2686466

[acel12481-bib-0022] Johnston TP (2004) The P‐407‐induced murine model of dose‐controlled hyperlipidemia and atherosclerosis: a review of findings to date. J. Cardiovasc. Pharmacol. 43, 595–606. 15085072 10.1097/00005344-200404000-00016

[acel12481-bib-0023] Kanehisa M , Goto S (2000) KEGG: kyoto encyclopedia of genes and genomes. Nucleic Acids Res. 28, 27–30. 10592173 10.1093/nar/28.1.27 PMC102409

[acel12481-bib-0024] Kanehisa M , Goto S , Sato Y , Kawashima M , Furumichi M , Tanabe M (2014) Data, information, knowledge and principle: back to metabolism in KEGG. Nucleic Acids Res. 42, D199–D205. 24214961 10.1093/nar/gkt1076 PMC3965122

[acel12481-bib-0025] Kawaguchi T , Taniguchi E , Itou M , Sakata M , Sumie S , Sata M (2011) Insulin resistance and chronic liver disease. World J. Hepatol. 3, 99–107. 21731901 10.4254/wjh.v3.i5.99 PMC3124882

[acel12481-bib-0026] Kotronen A , Vehkavaara S , Seppala‐Lindroos A , Bergholm R , Yki‐Jarvinen H (2007) Effect of liver fat on insulin clearance. Am. J. Physiol. Endocrinol. Metab. 293, E1709–E1715. 17895288 10.1152/ajpendo.00444.2007

[acel12481-bib-0027] Le Couteur DG , Cogger VC , Markus AMA , Harvey PJ , Yin ZL , Ansselin AD , McLean AJ (2001) Pseudocapillarization and associated energy limitation in the aged rat liver. Hepatology 33, 537–543. 11230732 10.1053/jhep.2001.22754

[acel12481-bib-0028] Le Couteur DG , Fraser R , Hilmer S , Rivory LP , McLean AJ (2005) The hepatic sinusoid in aging and cirrhosis: effects on hepatic substrate disposition and drug clearance. Clin. Pharmacokinet. 44, 187–200. 15656697 10.2165/00003088-200544020-00004

[acel12481-bib-0029] Le Couteur DG , Warren A , Cogger VC , Smedsrod B , Sorensen KK , De Cabo R , Fraser R , McCuskey RS (2008) Old age and the hepatic sinusoid. Anat. Rec. (Hoboken) 291, 672–683. 18484614 10.1002/ar.20661

[acel12481-bib-0030] Majumdar S , Genders AJ , Inyard AC , Frison V , Barrett EJ (2012) Insulin entry into muscle involves a saturable process in the vascular endothelium. Diabetologia 55, 450–456. 22002008 10.1007/s00125-011-2343-x PMC3270327

[acel12481-bib-0031] McMahon AC , Parry SN , Benson VL , Witting PK , Le Couteur DG (2013) Beneficial effects of the synthetic antioxidant tert‐butyl bisphenol on the hepatic microcirculation in a rat model of diabetes mellitus. Acta Diabetol. 50, 645–649. 22183926 10.1007/s00592-011-0358-x

[acel12481-bib-0032] Michael MD , Kulkarni RN , Postic C , Previs SF , Shulman GI , Magnuson MA , Kahn CR (2000) Loss of insulin signaling in hepatocytes leads to severe insulin resistance and progressive hepatic dysfunction. Mol. Cell 6, 87–97. 10949030

[acel12481-bib-0033] Mitchell SJ , Huizer‐Pajkos A , Cogger VC , McLachlan AJ , Le Couteur DG , Hilmer SN (2011) Poloxamer 407 increases the recovery of paracetamol in the isolated perfused rat liver. J. Pharm. Sci. 100, 334–340. 20564335 10.1002/jps.22235

[acel12481-bib-0034] Mitchell SJ , Huizer‐Pajkos A , Cogger VC , McLachlan AJ , Le Couteur DG , Jones B , de Cabo R , Hilmer SN (2012) The influence of old age and poloxamer‐407 on the hepatic disposition of diazepam in the isolated perfused rat liver. Pharmacology 90, 233–241. 23007459 10.1159/000341724 PMC5976489

[acel12481-bib-0035] Muller MJ , Willmann O , Rieger A , Fenk A , Selberg O , Lautz HU , Burger M , Balks HJ , von zMA , Schmidt FW (1992) Mechanism of insulin resistance associated with liver cirrhosis. Gastroenterology 102, 2033–2041. 1587421 10.1016/0016-5085(92)90329-w

[acel12481-bib-0036] Oya J , Nakagami T , Yamamoto Y , Fukushima S , Takeda M , Endo Y , Uchigata Y (2014) Effects of age on insulin resistance and secretion in subjects without diabetes. Int. Med. 53, 941–947. 10.2169/internalmedicine.53.1580 24785884

[acel12481-bib-0037] Philippe J , Halban PA , Gjinovci A , Duckworth WC , Estreicher J , Renold AE (1981) Increased clearance and degradation of [3H]insulin in streptozotocin diabetic rats. J. Clin. Investig. 67, 673–680. 6451633 10.1172/JCI110082 PMC370616

[acel12481-bib-0038] Rojas JM , Schwartz MW (2014) Control of hepatic glucose metabolism by islet and brain. Diabetes Obes. Metab. 16(Suppl 1), 33–40. 25200294 10.1111/dom.12332 PMC4191916

[acel12481-bib-0039] Sandqvist M , Strindberg L , Schmelz M , Lonnroth P , Jansson PA (2011) Impaired delivery of insulin to adipose tissue and skeletal muscle in obese women with postprandial hyperglycemia. J. Clin. Endocrinol. Metab. 96, E1320–E1324. 21677042 10.1210/jc.2011-0233

[acel12481-bib-0040] Sodoyez JC , Sodoyez‐Goffaux FR , Moris YM (1980) 125I‐insulin: kinetics of interaction with its receptors and rate of degradation in vivo. Am. J. Physiol. 239, E3–E8. 6994507 10.1152/ajpendo.1980.239.1.E3

[acel12481-bib-0041] Szklarczyk D , Franceschini A , Kuhn M , Simonovic M , Roth A , Minguez P , Doerks T , Stark M , Muller J , Bork P , Jensen LJ , von Mering C (2011) The STRING database in 2011: functional interaction networks of proteins, globally integrated and scored. Nucleic Acids Res. 39, D561–D568. 21045058 10.1093/nar/gkq973 PMC3013807

[acel12481-bib-0042] Taguchi K , Yamanaka‐Okumura H , Mizuno A , Nakamura T , Shimada M , Doi T , Takeda E (2014) Insulin resistance as early sign of hepatic dysfunction in liver cirrhosis. J. Med. Invest. 61, 180–189. 24705764 10.2152/jmi.61.180

[acel12481-bib-0043] Takamura T , Misu H , Ota T , Kaneko S (2012) Fatty liver as a consequence and cause of insulin resistance: lessons from type 2 diabetic liver. Endocr. J. 59, 745–763. 22893453 10.1507/endocrj.ej12-0228

[acel12481-bib-0044] Turner N , Kowalski GM , Leslie SJ , Risis S , Yang C , Lee‐Young RS , Babb JR , Meikle PJ , Lancaster GI , Henstridge DC , White PJ , Kraegen EW , Marette A , Cooney GJ , Febbraio MA , Bruce CR (2013) Distinct patterns of tissue‐specific lipid accumulation during the induction of insulin resistance in mice by high‐fat feeding. Diabetologia 56, 1638–1648. 23620060 10.1007/s00125-013-2913-1

[acel12481-bib-0045] Warren A , Benseler V , Cogger VC , Bertolino P , Le Couteur DG (2011) The impact of poloxamer 407 on the ultrastructure of the liver and evidence for clearance by extensive endothelial and kupffer cell endocytosis. Toxicol. Pathol. 39, 390–397. 21257999 10.1177/0192623310394212

[acel12481-bib-0046] Wisse W , De Zanger RB , Charels K , Van Der Smissen P , McCuskey RS (1985) The liver sieve: considerations concerning the structure and function of endothelial fenestrae, the sinusoidal wall and the space of Disse. Hepatology 5, 683–692. 3926620 10.1002/hep.1840050427

[acel12481-bib-0047] Xu N , Chow A , Goresky CA , Pang KS (1990) Effects of retrograde flow on measured blood volume, Disse space, intracellular water space and drug extraction in the perfused rat liver: characterization by the multiple indicator dilution technique. J. Pharmacol. Exp. Ther. 254, 914–925. 2395120

