# Supplementary material for: Ultrastructure of the liver microcirculation influences hepatic and systemic insulin activity and provides a mechanism for age‐related insulin resistance
Source: Aging Cell. 2016 Apr 20;15(4):706–15. doi: 10.1111/acel.12481 (PMC4933657; doi:10.1111/acel.12481)

S1 H&E Staining of liver tissue from the young and old rats and control and P07 treated rats. There was no evidence of liver pathology in any of the animal groups (Scale bar = 1 micron)

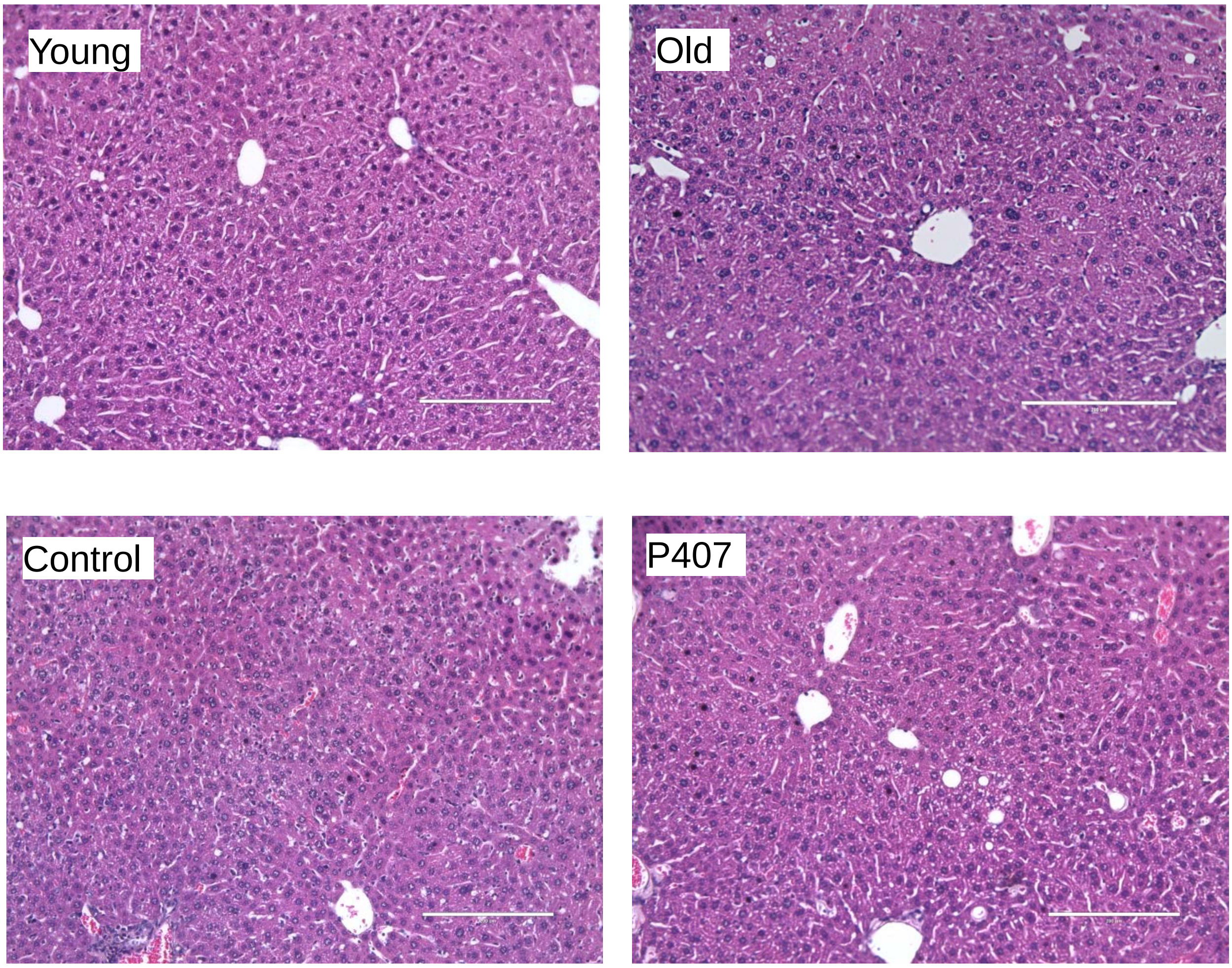

S2 Multiple Indicator Dilution experiments in middle aged F344 rats. There was an intermediate loss of insulin transfer in the middle aged rats.

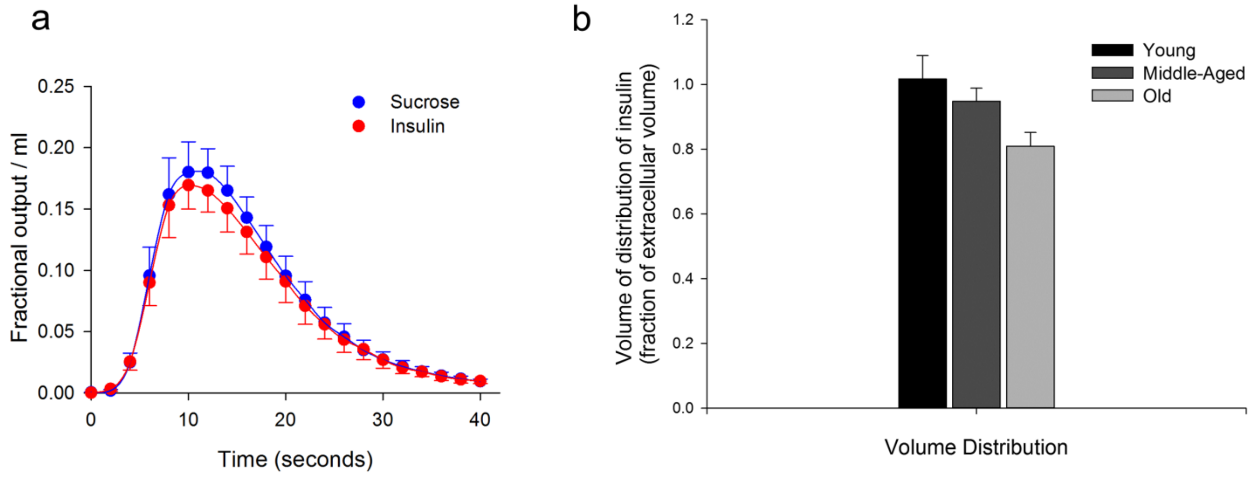

S3 Supplementary information for Phosphoproteomics

Quantitative phosphoproteomics of 47 peptides containing insulin-regulated sites from the KEGG insulin signaling pathway. Z-scores of >+/-1.0 were considered significant. Log2 values are provided to show statistical variation.

z-score (P407 v. control) | *z*-score (Old v. young)

Log_2 ratio (P407 v. control) | Log_2 ratio (Old v. young)

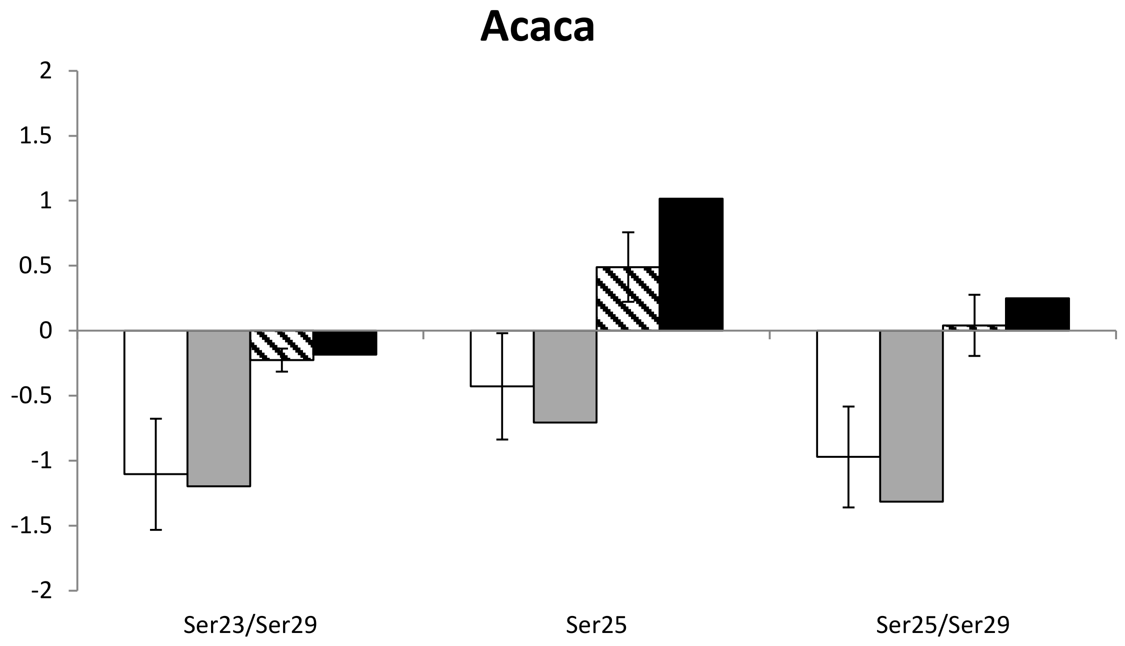

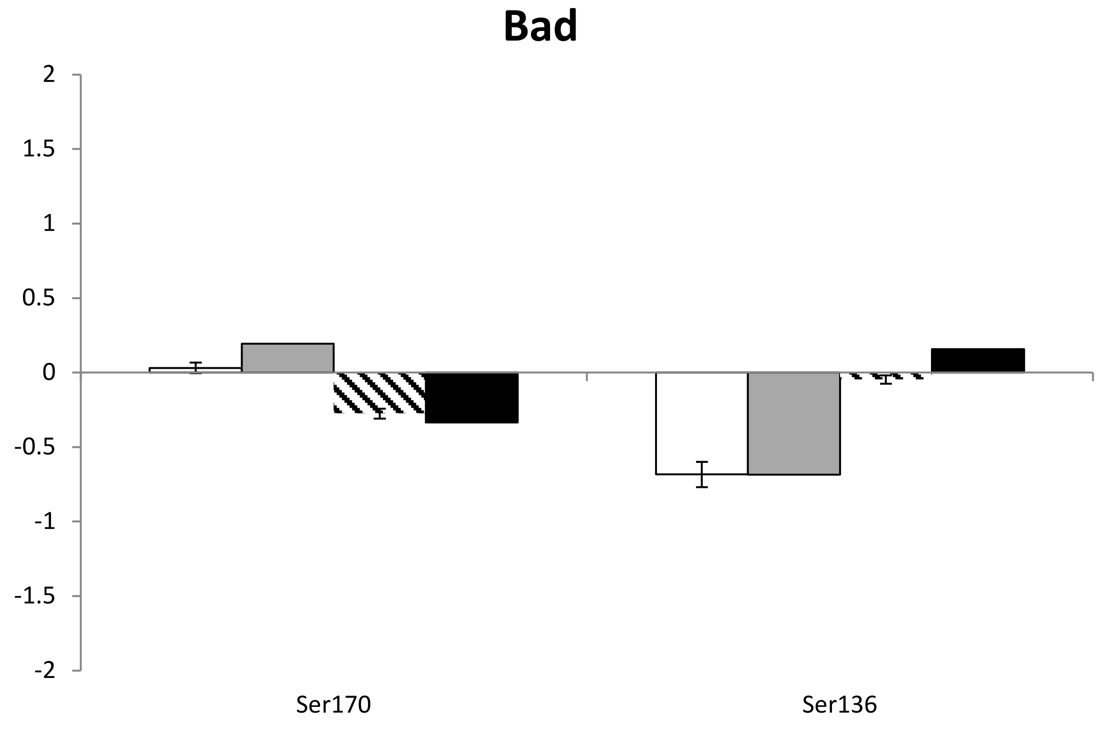

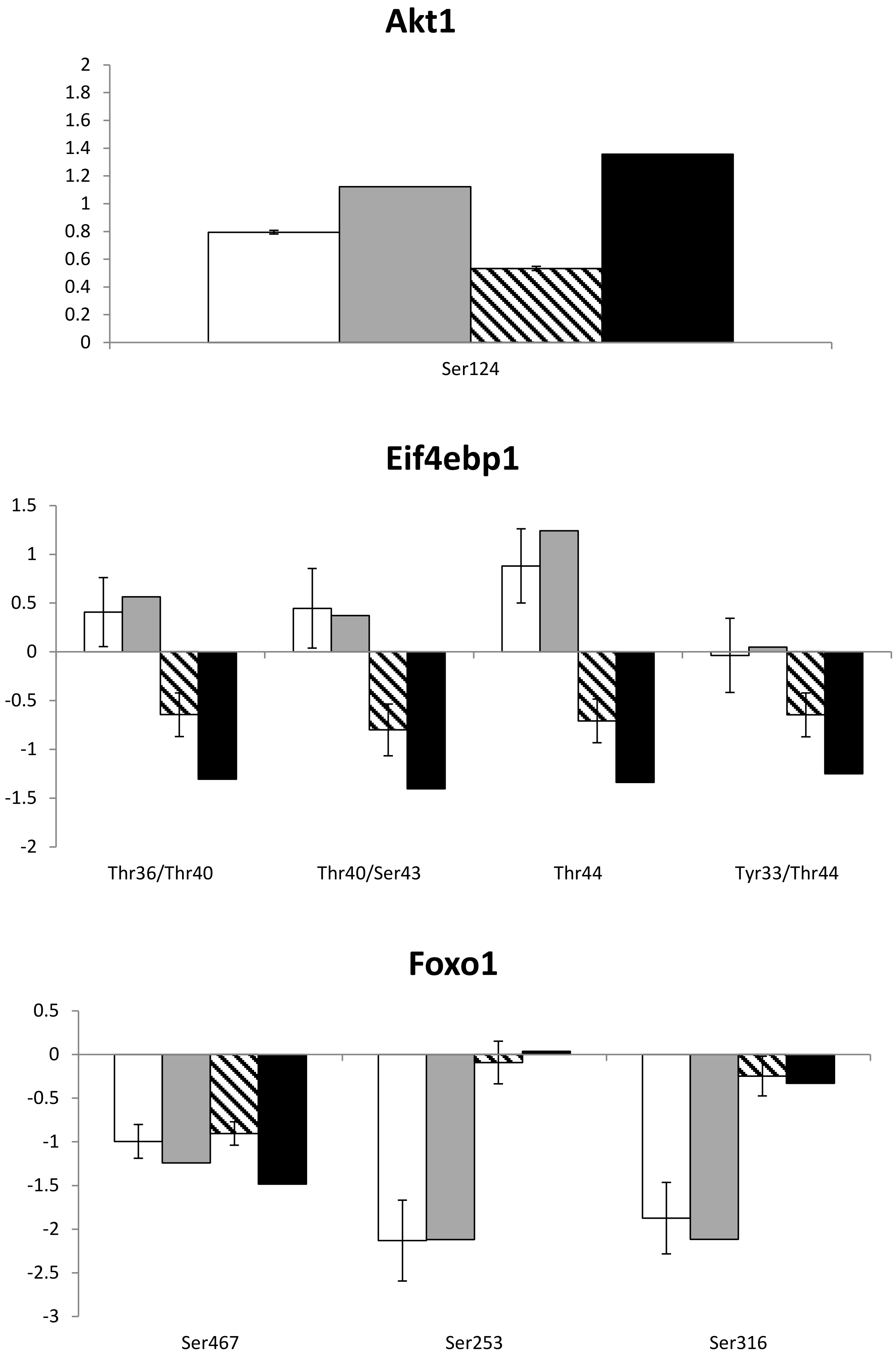
Akt1
2
1.8
1.6
1.4
1.2
1
0.8
0.6
0.4
0.2
0
Ser124
Eif4ebp1
1.5
1
0.5
0
-0.5
-1
-1.5
-2
Thr36/Thr40
Thr40/Ser43
Thr44
Tyr33/Thr44
Foxo1
0.5
0
-0.5
-1
-1.5
-2
-2.5
-3
Ser467
Ser253
Ser316

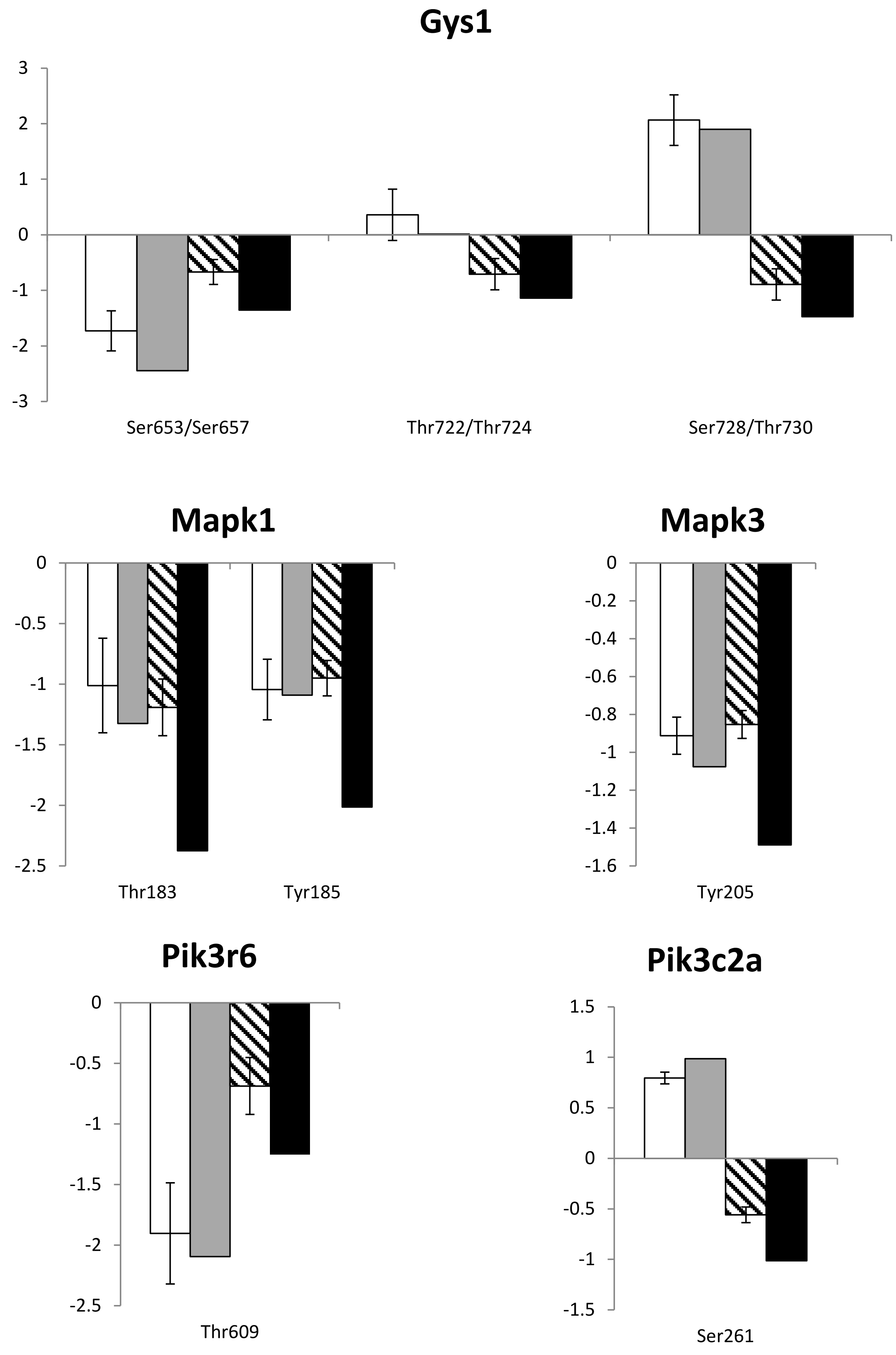
Gys1
3
2
1
0
-1
-2
-3
Ser653/Ser657
Thr722/Thr724
Ser728/Thr730
Mapk1
0
-0.5
-1
-1.5
-2
-2.5
Thr183
Tyr185
Mapk3
0
-0.2
-0.4
-0.6
-0.8
-1
-1.2
-1.4
-1.6
Tyr205
Pik3r6
0
-0.5
-1
-1.5
-2
-2.5
Thr609
Pik3c2a
1.5
1
0.5
0
-0.5
-1
-1.5
Ser261

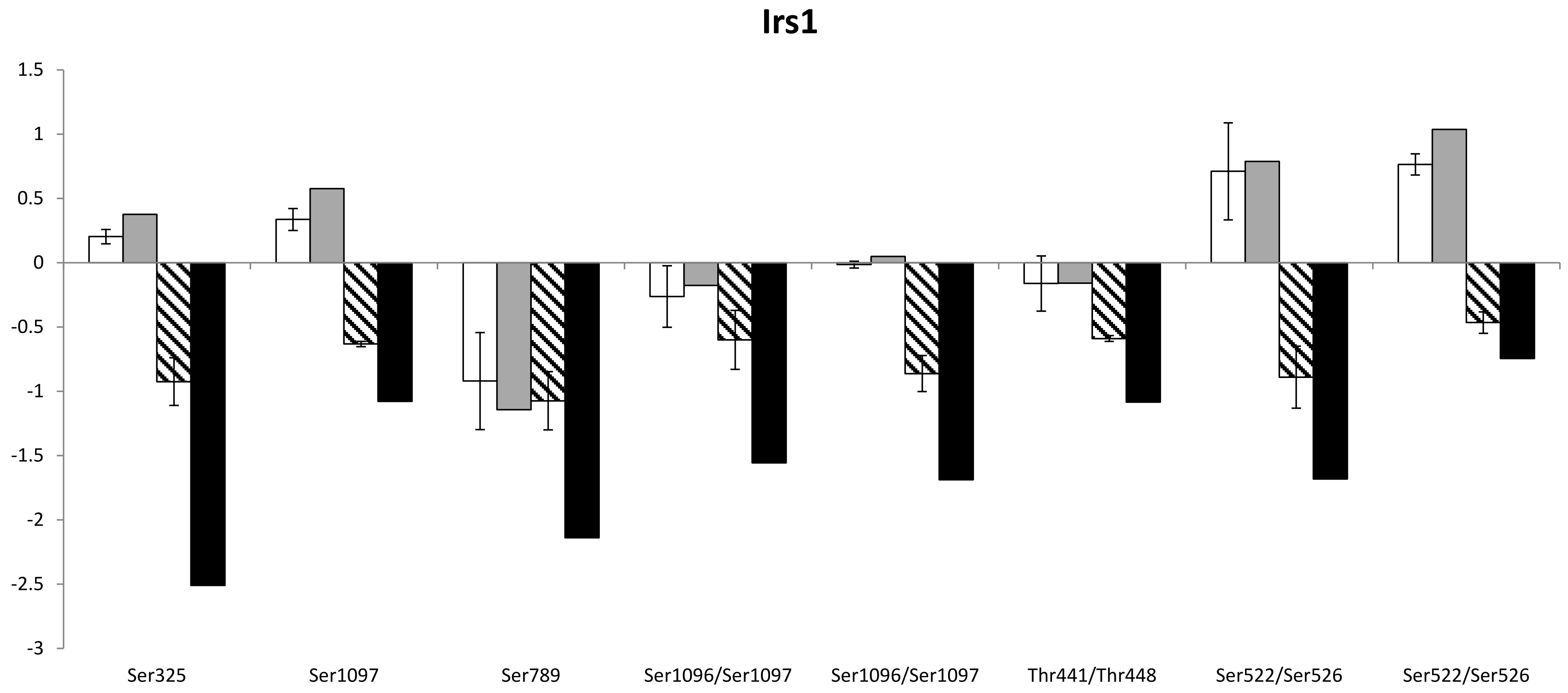
Irs1
1.5
1
0.5
0
-0.5
-1
-1.5
-2
-2.5
-3
Ser325
Ser1097
Ser789
Ser1096/Ser1097
Ser1096/Ser1097
Thr441/Thr448
Ser522/Ser526
Ser522/Ser526

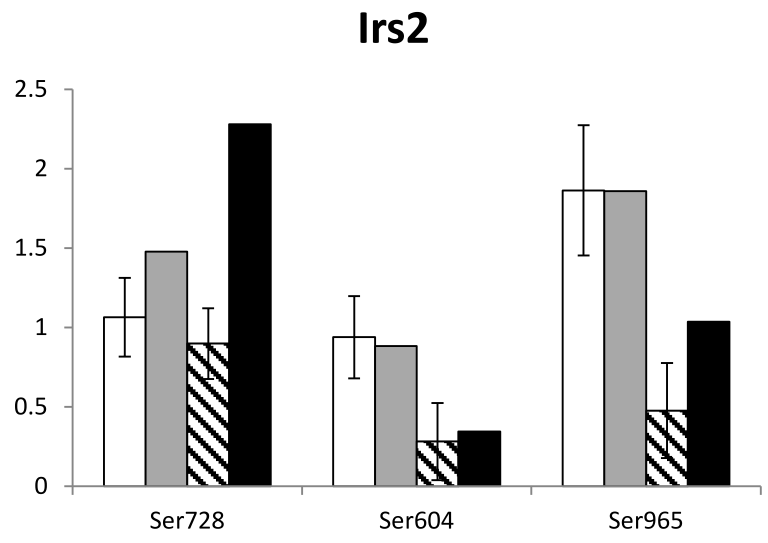
Irs2
2.5
2
1.5
1
0.5
0
Ser728
Ser604
Ser965

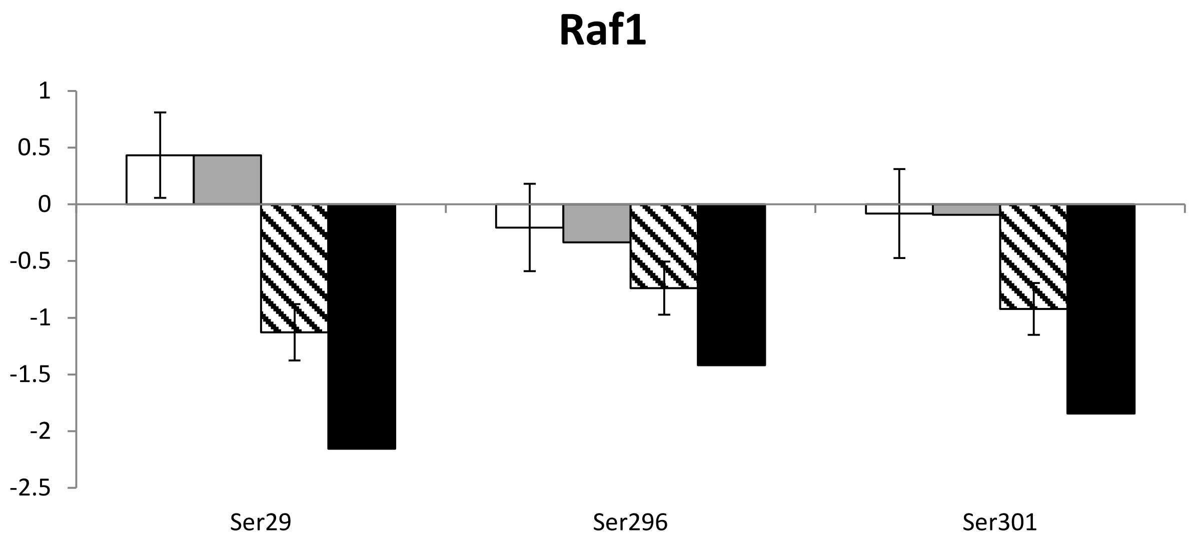
Raf1
1
0.5
0
-0.5
-1
-1.5
-2
-2.5
Ser29
Ser296
Ser301

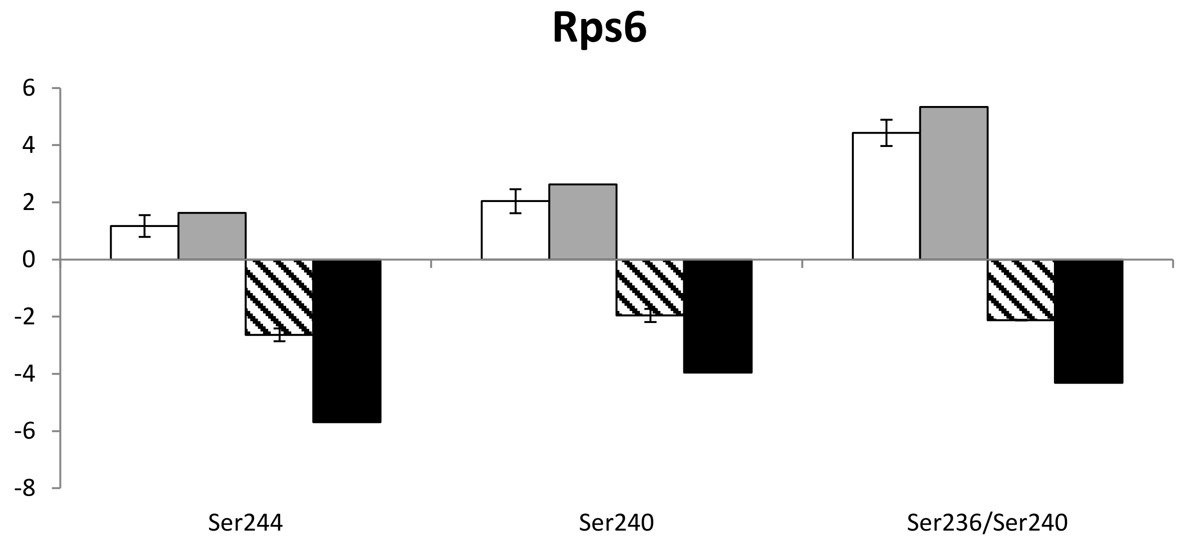
Rps6
6
4
2
0
-2
-4
-6
-8
Ser244
Ser240
Ser236/Ser240

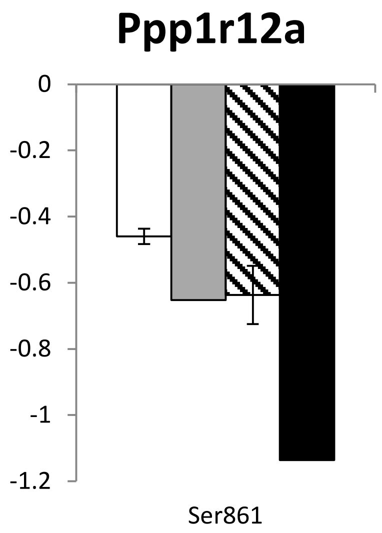
Ppp1r12a
0
-0.2
-0.4
-0.6
-0.8
-1
-1.2
Ser861

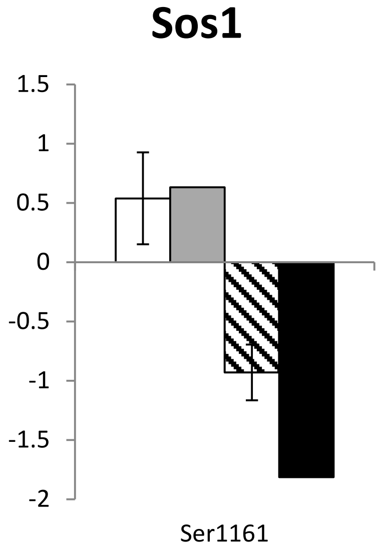
Sos1
1.5
1
0.5
0
-0.5
-1
-1.5
-2
Ser1161

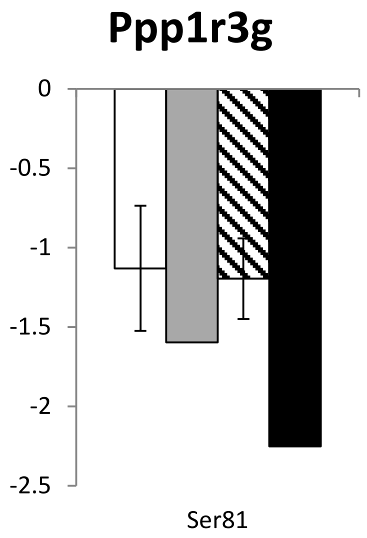
Ppp1r3g
0
-0.5
-1
-1.5
-2
-2.5
Ser81

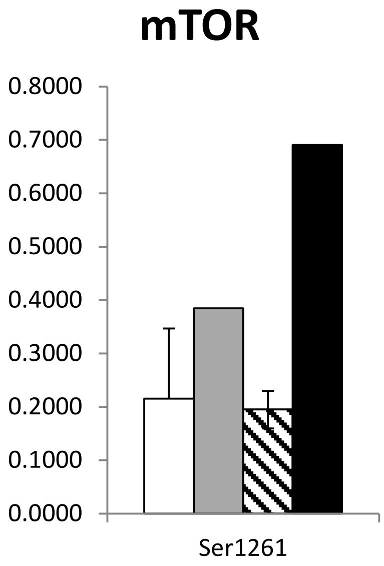
mTOR
0.8000
0.7000
0.6000
0.5000
0.4000
0.3000
0.2000
0.1000
0.0000
Ser1261

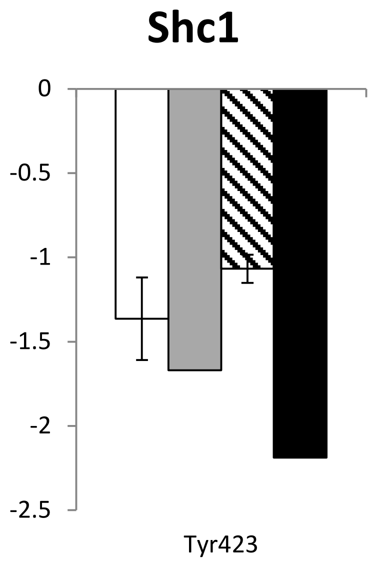
Shc1
0
-0.5
-1
-1.5
-2
-2.5
Tyr423

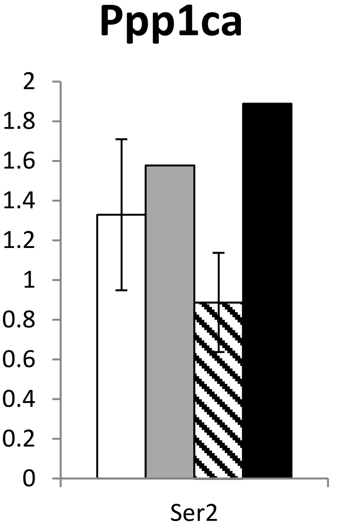
Ppp1ca
2
1.8
1.6
1.4
1.2
1
0.8
0.6
0.4
0.2
0
Ser2

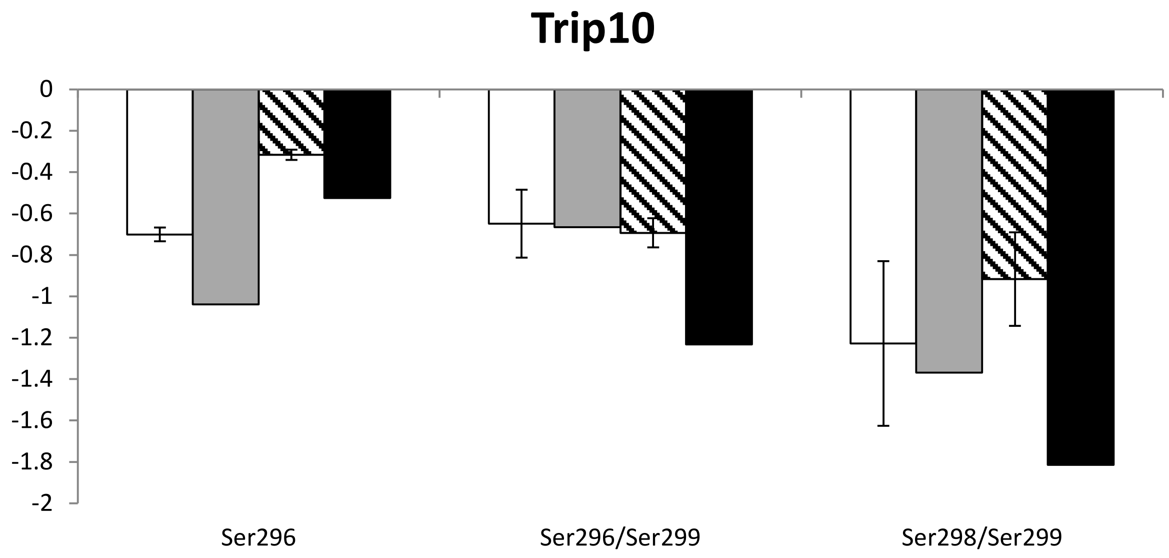
Trip10
0
-0.2
-0.4
-0.6
-0.8
-1
-1.2
-1.4
-1.6
-1.8
-2
Ser296
Ser296/Ser299
Ser298/Ser299

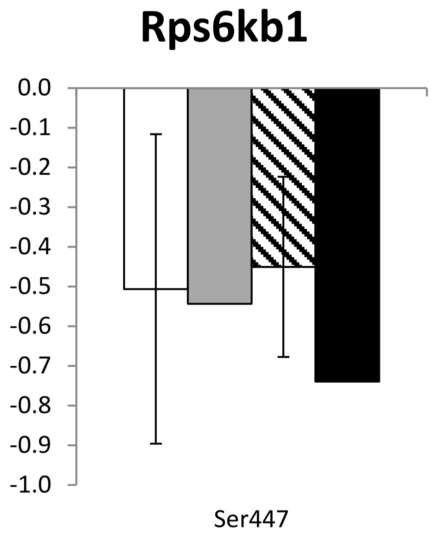
Rps6kb1
0.0
-0.1
-0.2
-0.3
-0.4
-0.5
-0.6
-0.7
-0.8
-0.9
-1.0
Ser447

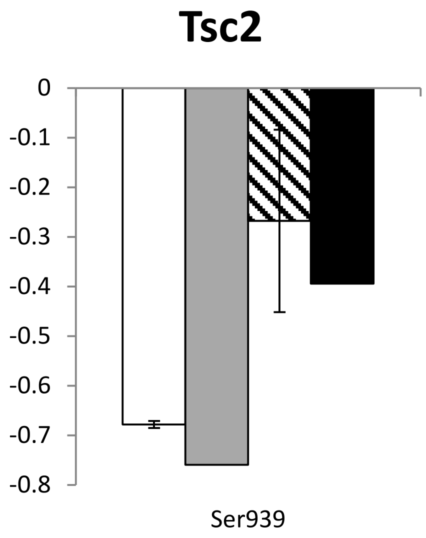
Tsc2
0
-0.1
-0.2
-0.3
-0.4
-0.5
-0.6
-0.7
-0.8
Ser939

S4- RTPCR data for Insulin signalling pathway in Young versus Old livers and Control versus P407 livers

Legend:
Fold-Change (2^(- Delta Delta Ct)) is the normalized gene expression (2^(- Delta Ct)) in the Test Sample divided the normalized gene expression (2^(- Delta Ct)) in the Control Sample.
Fold-Regulation represents fold-change results in a biologically meaningful way. Fold-change values greater than one indicate a positive- or an up-regulation, and the fold-regulation is equal to the fold-change
Fold-change values less than one indicate a negative or down-regulation, and the fold-regulation is the negative inverse of the fold-change.
Fold-change and fold-regulation values greater than 2 are indicated in red; fold-change values less than 0.5 and fold-regulation values less than -2 are indicated in blue.
p-values: The p values are calculated based on a Student's t-test of the replicate 2^(- Delta Ct) values for each gene in the control group and treatment groups, and p values less than 0.05 are indicated in red.
Comments:
A: This gene's average threshold cycle is relatively high (> 30) in either the control or the test sample, and is reasonably low in the other sample (< 30).
These data mean that the gene's expression is relatively low in one sample and reasonably detected in the other sample suggesting that the actual fold-change value is at least as large as the calculated and reported fold-change result.
This fold-change result may also have greater variations if p value > 0.05; therefore, it is important to have a sufficient number of biological replicates to validate the result for this gene.
B: This gene's average threshold cycle is relatively high (> 30), meaning that its relative expression level is low, in both control and test samples, and the p-value for the fold-change is either unavailable or relatively high (p > 0.05).
This fold-change result may also have greater variations; therefore, it is important to have a sufficient number of biological replicates to validate the result for this gene.
C: This gene's average threshold cycle is either not determined or greater than the defined cut-off (deault 35), in both samples meaning that its expression was undetected, making this fold-change result erroneous and un-interpretable.

Symbol	Well	AVG ΔC_t (Ct(GOI) - Ave Ct (HKG))		2^-ΔC_t		Fold Change	T-TEST	Fold Up- or Down-Regulation
		OLD	YOUNG	OLD	YOUNG	OLD /YOUNG	p value	OLD /YOUNG
Acaca	A01	3.11	2.33	1.2E-01	2.0E-01	0.58	0.294059	-1.71
Acox1	A02	5.05	6.38	3.0E-02	1.2E-02	2.52	0.965517	2.52
Adra1d	A03	9.45	10.32	1.4E-03	7.8E-04	1.83	0.342633	1.83
Aebp1	A04	7.88	6.60	4.2E-03	1.0E-02	0.41	0.402924	-2.42
Akt1	A05	4.15	4.00	5.6E-02	6.3E-02	0.90	0.814608	-1.11
Akt2	A06	6.83	8.03	8.8E-03	3.8E-03	2.30	0.863440	2.30
Akt3	A07	7.14	7.38	7.1E-03	6.0E-03	1.18	0.600176	1.18
Araf	A08	2.64	2.74	1.6E-01	1.5E-01	1.08	0.685440	1.08
Bcl2l1	A09	6.30	5.66	1.3E-02	2.0E-02	0.64	0.511245	-1.55
Braf	A10	5.00	5.19	3.1E-02	2.7E-02	1.14	0.961619	1.14
Cap1	A11	2.44	3.16	1.8E-01	1.1E-01	1.65	0.322493	1.65
Cbl	A12	7.53	7.76	5.4E-03	4.6E-03	1.17	0.952396	1.17
Cebpa	B01	2.33	2.18	2.0E-01	2.2E-01	0.91	0.732909	-1.10
Cebpb	B02	6.27	7.75	1.3E-02	4.6E-03	2.80	0.996637	2.80
Cfd	B03	7.67	8.33	4.9E-03	3.1E-03	1.58	0.539415	1.58
Dok1	B04	9.19	10.19	1.7E-03	8.5E-04	2.01	0.341850	2.01
Dok2	B05	9.17	9.79	1.7E-03	1.1E-03	1.53	0.394094	1.53
Dok3	B06	9.31	9.86	1.6E-03	1.1E-03	1.46	0.421388	1.46
Dusp14	B07	8.27	7.45	3.2E-03	5.7E-03	0.57	0.780596	-1.76
Eif2b1	B08	4.71	4.08	3.8E-02	5.9E-02	0.64	0.272332	-1.55
Eif4ebp1	B09	1.85	1.24	2.8E-01	4.2E-01	0.66	0.306678	-1.53
Ercc1	B10	7.74	9.01	4.7E-03	1.9E-03	2.42	0.801794	2.42
Fasn	B11	3.80	-0.91	7.2E-02	1.9E+00	**0.04**	**0.022161**	**-26.22**
Fbp1	B12	8.02	8.14	3.9E-03	3.5E-03	1.09	0.859602	1.09
Fos	C01	7.14	8.67	7.1E-03	2.5E-03	2.90	0.789263	2.90
Frs2	C02	6.62	6.94	1.0E-02	8.1E-03	1.25	0.505022	1.25
Frs3	C03	5.82	7.04	1.8E-02	7.6E-03	2.33	0.711226	2.33
G6pc	C04	7.95	6.89	4.0E-03	8.4E-03	0.48	0.672318	-2.08
Gab1	C05	5.29	5.70	2.6E-02	1.9E-02	1.32	0.796305	1.32
Gcg	C06	6.57	7.70	1.1E-02	4.8E-03	2.18	0.659937	2.18
Gck	C07	6.22	6.58	1.3E-02	1.0E-02	1.28	0.789675	1.28
Gpd1	C08	7.75	7.34	4.6E-03	6.2E-03	0.75	0.542770	-1.33
Grb10	C09	6.06	6.65	1.5E-02	1.0E-02	1.51	0.698559	1.51
Grb2	C10	6.82	8.10	8.8E-03	3.7E-03	2.42	0.753414	2.42
Gsk3b	C11	8.84	10.07	2.2E-03	9.3E-04	2.34	0.324282	2.34
Hk2	C12	6.37	7.40	1.2E-02	5.9E-03	2.04	0.594500	2.04
Hras	D01	5.97	6.82	1.6E-02	8.9E-03	1.80	0.448238	1.80
Igf1r	D02	9.29	8.83	1.6E-03	2.2E-03	0.73	0.928575	-1.37

Symbol	Well	AVG ΔC$_t$ (Ct(GOI) - Ave Ct (HKG))		2^-ΔC$_t$		Fold Change	T-TEST	Fold Up- or Down-Regulation
		OLD	YOUNG	OLD	YOUNG	OLD /YOUNG	p value	OLD /YOUNG
Igf2	D03	4.72	6.40	3.8E-02	1.2E-02	3.21	0.822011	3.21
Igfbp1	D04	9.45	9.57	1.4E-03	1.3E-03	1.09	0.477142	1.09
Ins1	D05	9.45	10.32	1.4E-03	7.8E-04	1.83	0.342633	1.83
Ins2	D06	7.44	9.41	5.8E-03	1.5E-03	3.93	0.196303	3.93
Insl3	D07	6.61	8.45	1.0E-02	2.9E-03	3.60	0.598694	3.60
Insr	D08	2.46	2.86	1.8E-01	1.4E-01	1.32	0.813029	1.32
Irs1	D09	3.77	3.50	7.3E-02	8.9E-02	0.83	0.845756	-1.21
Irs2	D10	4.55	5.13	4.3E-02	2.8E-02	1.50	0.363243	1.50
Jun	D11	3.66	2.75	7.9E-02	1.5E-01	0.53	0.247933	-1.87
Klf10	D12	7.21	6.06	6.7E-03	1.5E-02	0.45	0.531329	-2.23
Kras	E01	4.77	4.76	3.7E-02	3.7E-02	0.99	0.756316	-1.01
Ldlr	E02	6.48	7.70	1.1E-02	4.8E-03	2.33	0.728540	2.33
Lep	E03	7.17	8.40	6.9E-03	3.0E-03	2.34	0.817543	2.34
Map2k1	E04	3.11	3.09	1.2E-01	1.2E-01	0.99	0.728693	-1.01
Mapk1	E05	4.74	4.46	3.7E-02	4.6E-02	0.82	0.748205	-1.22
Mtor	E06	9.45	10.32	1.4E-03	7.8E-04	1.83	0.342633	1.83
Nos2	E07	7.70	8.94	4.8E-03	2.0E-03	2.36	0.898031	2.36
Npy	E08	7.33	8.50	6.2E-03	2.8E-03	2.26	0.880665	2.26
Pck2	E09	6.22	5.92	1.3E-02	1.7E-02	0.81	0.712001	-1.23
Pdpk1	E10	4.94	4.86	3.3E-02	3.4E-02	0.94	0.950127	-1.06
Pik3ca	E11	6.87	8.17	8.5E-03	3.5E-03	2.47	0.846339	2.47
Pik3cb	E12	8.86	10.32	2.1E-03	7.8E-04	2.74	0.296042	2.74
Pik3r1	F01	3.18	3.15	1.1E-01	1.1E-01	0.98	0.692093	-1.02
Pik3r2	F02	5.97	5.30	1.6E-02	2.5E-02	0.63	0.464881	-1.59
Pklr	F03	6.24	4.80	1.3E-02	3.6E-02	0.37	0.464414	-2.71
Pparg	F04	9.45	9.94	1.4E-03	1.0E-03	1.41	0.404963	1.41
Ppp1ca	F05	2.65	2.72	1.6E-01	1.5E-01	1.05	0.961231	1.05
Prkcg	F06	7.51	8.98	5.5E-03	2.0E-03	2.78	0.660055	2.78
Prkcz	F07	7.05	6.57	7.5E-03	1.0E-02	0.72	0.980219	-1.39
Prl	F08	7.20	8.70	6.8E-03	2.4E-03	2.82	0.811913	2.82
Ptpn1	F09	6.93	8.58	8.2E-03	2.6E-03	3.14	0.713847	3.14
Raf1	F10	1.89	2.55	2.7E-01	1.7E-01	1.58	0.363834	1.58
Retn	F11	9.45	10.32	1.4E-03	7.8E-04	1.83	0.342633	1.83
Rps6ka1	F12	7.87	9.15	4.3E-03	1.8E-03	2.43	0.661146	2.43
Rras	G01	4.90	4.07	3.4E-02	6.0E-02	0.56	0.477690	-1.78
Rras2	G02	3.97	4.70	6.4E-02	3.9E-02	1.66	0.329842	1.66
Serpine1	G03	5.59	5.51	2.1E-02	2.2E-02	0.94	0.806348	-1.06
Shc1	G04	5.09	5.11	2.9E-02	2.9E-02	1.01	0.729800	1.01

Symbol	Well	AVG ΔCt (Ct(GOI) - Ave Ct (HKG))		2^-ΔCt		Fold Change	T-TEST	Fold Up- or Down-Regulation
		OLD	YOUNG	OLD	YOUNG	OLD /YOUNG	p value	OLD /YOUNG
Slc27a4	G05	7.29	8.70	6.4E-03	2.4E-03	2.66	0.899783	2.66
Slc2a1	G06	7.26	8.72	6.5E-03	2.4E-03	2.76	0.835286	2.76
Slc2a4	G07	6.46	7.41	1.1E-02	5.9E-03	1.93	0.922519	1.93
Sos1	G08	6.28	5.85	1.3E-02	1.7E-02	0.74	0.568297	-1.34
Srebf1	G09	1.82	1.09	2.8E-01	4.7E-01	0.60	0.291791	-1.66
Tg	G10	9.45	10.21	1.4E-03	8.4E-04	1.70	0.355179	1.70
Ucp1	G11	9.26	10.32	1.6E-03	7.8E-04	2.08	0.333236	2.08
Vegfa	G12	6.48	7.75	1.1E-02	4.6E-03	2.42	0.782530	2.42
Actb	H01	-1.03	-1.43	2.0E+00	2.7E+00	0.76	0.658113	-1.31
B2m	H02	2.82	1.90	1.4E-01	2.7E-01	0.53	0.769787	-1.89
Hprt1	H03	-0.84	0.20	1.8E+00	8.7E-01	2.06	0.922424	2.06
Ldha	H04	0.42	0.03	7.5E-01	9.8E-01	0.77	0.809575	-1.30
Rplp1	H05	-1.36	-0.71	2.6E+00	1.6E+00	1.57	0.388136	1.57

Symbol	Well	AVG ΔCt (Ct(GOI) - Ave Ct (HKG))		2^-ΔCt		Fold Change	T-TEST	Fold Up- or Down-Regulation
		P407	CONTROL	P407	CONTROL	P407 /CONTROL	p value	P407 /CONTROL
Acaca	A01	5.57	5.30	2.1E-02	2.5E-02	0.83	0.883138	-1.20
Acox1	A02	2.79	2.10	1.4E-01	2.3E-01	0.62	N/A	-1.62
Adra1d	A03	14.67	N/A	3.8E-05	N/A	N/A	N/A	N/A
Aebp1	A04	10.31	10.92	7.9E-04	5.2E-04	1.53	0.168876	1.53
Akt1	A05	5.86	6.12	1.7E-02	1.4E-02	1.20	0.555182	1.20
Akt2	A06	5.04	5.27	3.0E-02	2.6E-02	1.18	0.605222	1.18
Akt3	A07	9.65	9.65	1.2E-03	1.2E-03	1.00	0.917337	-1.00
Araf	A08	4.09	3.69	5.9E-02	7.7E-02	0.76	N/A	-1.31
Bcl2l1	A09	5.98	5.40	1.6E-02	2.4E-02	0.67	0.168497	-1.49
Braf	A10	7.66	7.80	4.9E-03	4.5E-03	1.10	0.511738	1.10
Cap1	A11	5.01	5.07	3.1E-02	3.0E-02	1.05	0.732696	1.05
Cbl	A12	9.68	10.05	1.2E-03	9.4E-04	1.30	0.407195	1.30
Cebpa	B01	3.67	6.27	7.9E-02	1.3E-02	6.08	0.950302	6.08
Cebpb	B02	12.79	12.65	1.4E-04	1.6E-04	0.90	N/A	-1.11
Cfd	B03	4.32	4.13	5.0E-02	5.7E-02	0.88	0.706524	-1.14
Dok1	B04	10.94	10.94	5.1E-04	5.1E-04	1.00	0.607590	1.00
Dok2	B05	12.17	12.12	2.2E-04	2.2E-04	0.97	0.722672	-1.03
Dok3	B06	10.00	11.15	9.7E-04	4.4E-04	2.21	0.083272	2.21
Dusp14	B07	9.72	9.60	1.2E-03	1.3E-03	0.92	0.577725	-1.08
Eif2b1	B08	7.00	7.31	7.8E-03	6.3E-03	1.24	N/A	1.24
Eif4ebp1	B09	4.97	4.98	3.2E-02	3.2E-02	1.00	0.966788	1.00
Ercc1	B10	11.91	11.99	2.6E-04	2.5E-04	1.06	0.874044	1.06
Fasn	B11	3.81	4.29	7.1E-02	5.1E-02	1.40	N/A	1.40
Fbp1	B12	1.80	1.47	2.9E-01	3.6E-01	0.80	0.502627	-1.26
Fos	C01	10.57	10.77	6.6E-04	5.7E-04	1.15	0.745289	1.15
Frs2	C02	7.57	7.39	5.3E-03	5.9E-03	0.89	0.419211	-1.13
Frs3	C03	12.21	12.40	2.1E-04	1.8E-04	1.14	0.509824	1.14
G6pc	C04	4.98	3.17	3.2E-02	1.1E-01	0.28	0.841671	-3.53
Gab1	C05	8.92	8.99	2.1E-03	2.0E-03	1.05	0.909824	1.05
Gcg	C06	14.67	N/A	3.8E-05	N/A	N/A	N/A	N/A
Gck	C07	6.55	5.77	1.1E-02	1.8E-02	0.58	N/A	-1.72
Gpd1	C08	3.87	3.46	6.8E-02	9.1E-02	0.75	0.553015	-1.33
Grb10	C09	12.21	12.34	2.1E-04	1.9E-04	1.09	0.605081	1.09
Grb2	C10	3.71	3.83	7.6E-02	7.0E-02	1.09	N/A	1.09
Gsk3b	C11	6.79	6.93	9.1E-03	8.2E-03	1.11	0.726290	1.11
Hk2	C12	12.22	12.62	2.1E-04	1.6E-04	1.33	0.380887	1.33
Hras	D01	3.37	3.25	9.7E-02	1.1E-01	0.92	0.813262	-1.09
Igf1r	D02	11.65	11.63	3.1E-04	3.2E-04	0.98	0.863731	-1.02

Symbol	Well	AVG ΔC$_t$ (Ct(GOI) - Ave Ct (HKG))		2^-ΔC$_t$		Fold Change	T-TEST	Fold Up- or Down-Regulation
		P407	CONTROL	P407	CONTROL	P407 /CONTROL	p value	P407 /CONTROL
Igf2	D03	12.16	12.28	2.2E-04	2.0E-04	1.09	0.576069	1.09
Igfbp1	D04	2.69	2.49	1.5E-01	1.8E-01	0.87	0.931905	-1.15
Ins1	D05	11.76	13.16	2.9E-04	1.1E-04	2.63	N/A	2.63
Ins2	D06	12.79	13.16	1.4E-04	1.1E-04	1.29	N/A	1.29
Insl3	D07	11.97	12.65	2.5E-04	1.6E-04	1.60	N/A	1.60
Insr	D08	5.44	5.88	2.3E-02	1.7E-02	1.35	0.227271	1.35
Irs1	D09	5.58	5.47	2.1E-02	2.3E-02	0.92	0.655403	-1.09
Irs2	D10	8.80	9.62	2.2E-03	1.3E-03	1.76	**0.026492**	1.76
Jun	D11	6.91	7.43	8.3E-03	5.8E-03	1.44	0.441436	1.44
Klf10	D12	7.66	7.78	4.9E-03	4.6E-03	1.08	0.663021	1.08
Kras	E01	7.66	7.48	4.9E-03	5.6E-03	0.88	0.310540	-1.13
Ldlr	E02	5.63	6.27	2.0E-02	1.3E-02	1.55	0.278261	1.55
Lep	E03	14.67	N/A	3.8E-05	N/A	**N/A**	N/A	**N/A**
Map2k1	E04	6.25	6.31	1.3E-02	1.3E-02	1.05	0.967957	1.05
Mapk1	E05	3.93	3.98	6.6E-02	6.3E-02	1.03	0.755070	1.03
Mtor	E06	7.07	7.00	7.5E-03	7.8E-03	0.95	0.748041	-1.05
Nos2	E07	13.20	12.65	1.1E-04	1.6E-04	0.68	N/A	-1.46
Npy	E08	7.95	7.37	4.1E-03	6.0E-03	0.67	0.380978	-1.49
Pck2	E09	4.99	5.84	3.1E-02	1.8E-02	1.79	0.184082	1.79
Pdpk1	E10	6.53	6.55	1.1E-02	1.1E-02	1.01	N/A	1.01
Pik3ca	E11	6.84	6.98	8.7E-03	7.9E-03	1.10	0.689183	1.10
Pik3cb	E12	6.91	7.01	8.3E-03	7.8E-03	1.07	0.798322	1.07
Pik3r1	F01	6.65	5.81	1.0E-02	1.8E-02	0.56	0.188509	-1.79
Pik3r2	F02	8.40	8.53	3.0E-03	2.7E-03	1.09	0.804854	1.09
Pklr	F03	3.86	2.86	6.9E-02	1.4E-01	0.50	0.477493	-2.00
Pparg	F04	10.40	10.21	7.4E-04	8.5E-04	0.88	0.749064	-1.14
Ppp1ca	F05	3.57	3.67	8.4E-02	7.9E-02	1.07	0.633707	1.07
Prkcg	F06	12.43	12.62	1.8E-04	1.6E-04	1.14	0.587596	1.14
Prkcz	F07	8.73	8.95	2.4E-03	2.0E-03	1.16	0.775432	1.16
Prl	F08	12.79	12.62	1.4E-04	1.6E-04	0.89	0.757958	-1.13
Ptpn1	F09	7.38	7.69	6.0E-03	4.8E-03	1.24	0.562210	1.24
Raf1	F10	3.73	3.92	7.5E-02	6.6E-02	1.14	0.453272	1.14
Retn	F11	14.67	13.16	3.8E-05	1.1E-04	0.35	N/A	-2.85
Rps6ka1	F12	7.12	7.18	7.2E-03	6.9E-03	1.04	0.944111	1.04
Rras	G01	5.37	5.85	2.4E-02	1.7E-02	1.39	0.235829	1.39
Rras2	G02	5.35	6.16	2.4E-02	1.4E-02	1.75	N/A	1.75
Serpine1	G03	11.18	11.67	4.3E-04	3.1E-04	1.40	0.765379	1.40
Shc1	G04	5.22	5.56	2.7E-02	2.1E-02	1.27	0.643608	1.27

Symbol	Well	AVG ΔC_t (Ct(GOI) - Ave Ct (HKG))		$2^{-\Delta C_t}$		Fold Change	T-TEST	Fold Up- or Down-Regulation
		P407	CONTROL	P407	CONTROL	P407 /CONTROL	p value	P407 /CONTROL
Slc27a4	G05	7.79	7.78	4.5E-03	4.6E-03	0.99	0.956394	-1.01
Slc2a1	G06	11.55	10.78	3.3E-04	5.7E-04	0.59	0.262772	-1.70
Slc2a4	G07	12.79	13.16	1.4E-04	1.1E-04	1.29	N/A	1.29
Sos1	G08	6.78	6.66	9.1E-03	9.9E-03	0.92	0.898960	-1.09
Srebf1	G09	9.09	9.58	1.8E-03	1.3E-03	1.41	N/A	1.41
Tg	G10	11.90	10.99	2.6E-04	4.9E-04	0.53	**0.010198**	-1.88
Ucp1	G11	13.22	12.14	1.1E-04	2.2E-04	0.47	N/A	-2.11
Vegfa	G12	4.95	4.90	3.2E-02	3.4E-02	0.96	0.857308	-1.04
Actb	H01	0.68	0.92	6.3E-01	5.3E-01	1.19	0.551329	1.19
B2m	H02	-2.55	-2.89	5.8E+00	7.4E+00	0.79	0.059386	-1.27
Hprt1	H03	1.68	0.98	3.1E-01	5.1E-01	0.62	0.239562	-1.63
Ldha	H04	0.34	0.88	7.9E-01	5.4E-01	1.46	0.108750	1.46
Rplp1	H05	-0.15	0.10	1.1E+00	9.3E-01	1.19	0.175467	1.19

S5 Increased blood levels of cholesterol and triglycerides following P407 treatment

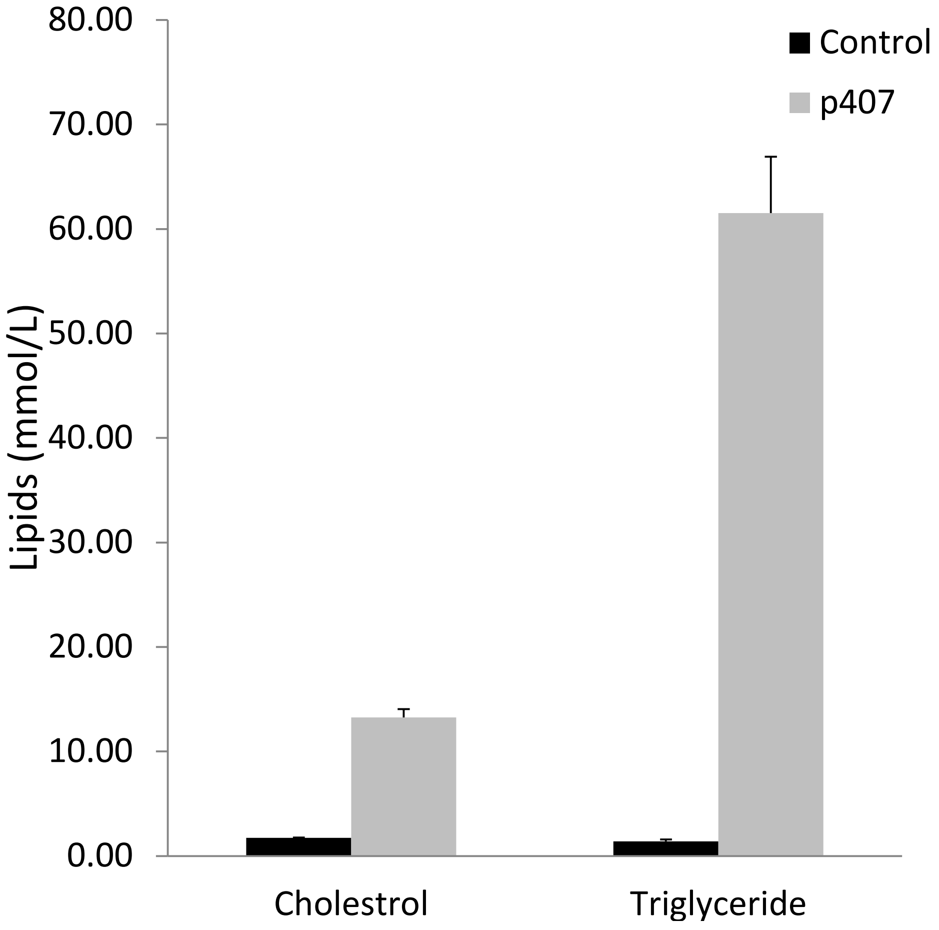

Supplement: Supplementary file 1 — Fig. S1 H&E Staining of liver tissue from the young and old rats and control and P07‐treated rats. Fig. S2 Insulin and sucrose curves and volume of distribution of insulin for the liver of middle aged F344 rats. Fig. S3 Supplementary information for phosphoproteomics. Fig. S4 RT–PCR data for Insulin signaling pathway in young versus old livers and control versus P407 livers. Fig. S5 Increased blood levels of cholesterol and triglycerides following P407 treatment. [file ACEL-15-706-s001.pdf]
